# Enhancing reproducibility in mixing time determination of stirred tank reactors via automated analysis and standardized inter-laboratory trials

**DOI:** 10.1007/s00253-026-13941-8

**Published:** 2026-06-30

**Authors:** Isabelle Barth, Jürgen Fitschen, Christian Sieblist, Ute Husemann, Dominic Kues, Christoph Neubrand, Christoph Bouvier, Cedric Schirmer, Bernd Tscheschke, Igor Vassilev, Percy Kampeis

**Affiliations:** 1 Institute for Biotechnical Process Design, Trier University of Applied Sciences, Environmental Campus Birkenfeld, Campusallee 9913, Hoppstädten-Weiersbach, 55768 Germany; 2 Boehringer Ingelheim Pharma GmbH & Co. KG, Biberach, Germany; 3 Roche Diagnostics GmbH, Penzberg, Germany; 4 Sartorius Stedim Biotech GmbH, Göttingen, Germany; 5 Rentschler Biopharma SE, Laupheim, Germany; 6 ZHAW Zurich University of Applied Sciences, Wädenswil, Switzerland; 7 Bayer AG, Leverkusen, Germany; 8 Eppendorf SE, Bioprocess Center, Juelich, Germany

**Keywords:** Stirred tank reactor, Fluid flow distribution, Turbulent shear forces, Homogenization, Local mixing time

## Abstract

**Abstract:**

Determining the mixing time in a mixing apparatus enables the evaluation of mixing quality and, therefore, represents a valuable tool for characterizing unit operations in process engineering. Mixing is highly relevant in both upstream processes (e.g., bioreactors) and downstream processes (e.g., blending tanks in diafiltration) in biotechnology and pharmaceutics. The aim of combining a colorimetric method with video capture and automated image analysis is to provide a robust, standardized methodology for determining mixing times in transparent bioreactors, such as laboratory-scale glass bioreactors and single-use bioreactors. A Python-based tool for video analysis was developed for this purpose. The Python script provides different mixing indices that enable the evaluation of both global and local mixing times under varying stirring speeds and aeration rates. This approach offers deep insights into the mixing process and enables the identification of heterogeneities. A round-robin study involving members of the DECHEMA Working Group “Single-Use Technologies for Bio-Based Applications” is currently being conducted to enhance the analysis and ensure reproducibility across different laboratories, thereby providing a robust basis for validating computational-fluid dynamics simulations. Initial reproducibility issues were identified and addressed in the standard operating procedure, demonstrating that a framework for automated analysis and improved standardization has been established.

**Key points:**

• *Mixing-time experiments conducted at different aeration rates and stirring speeds*

• *Automated video analysis for determining mixing times on both global and local scales*

• *Identification of heterogeneities through visualization of local mixing times*

**Graphical abstract:**

**Figure:**
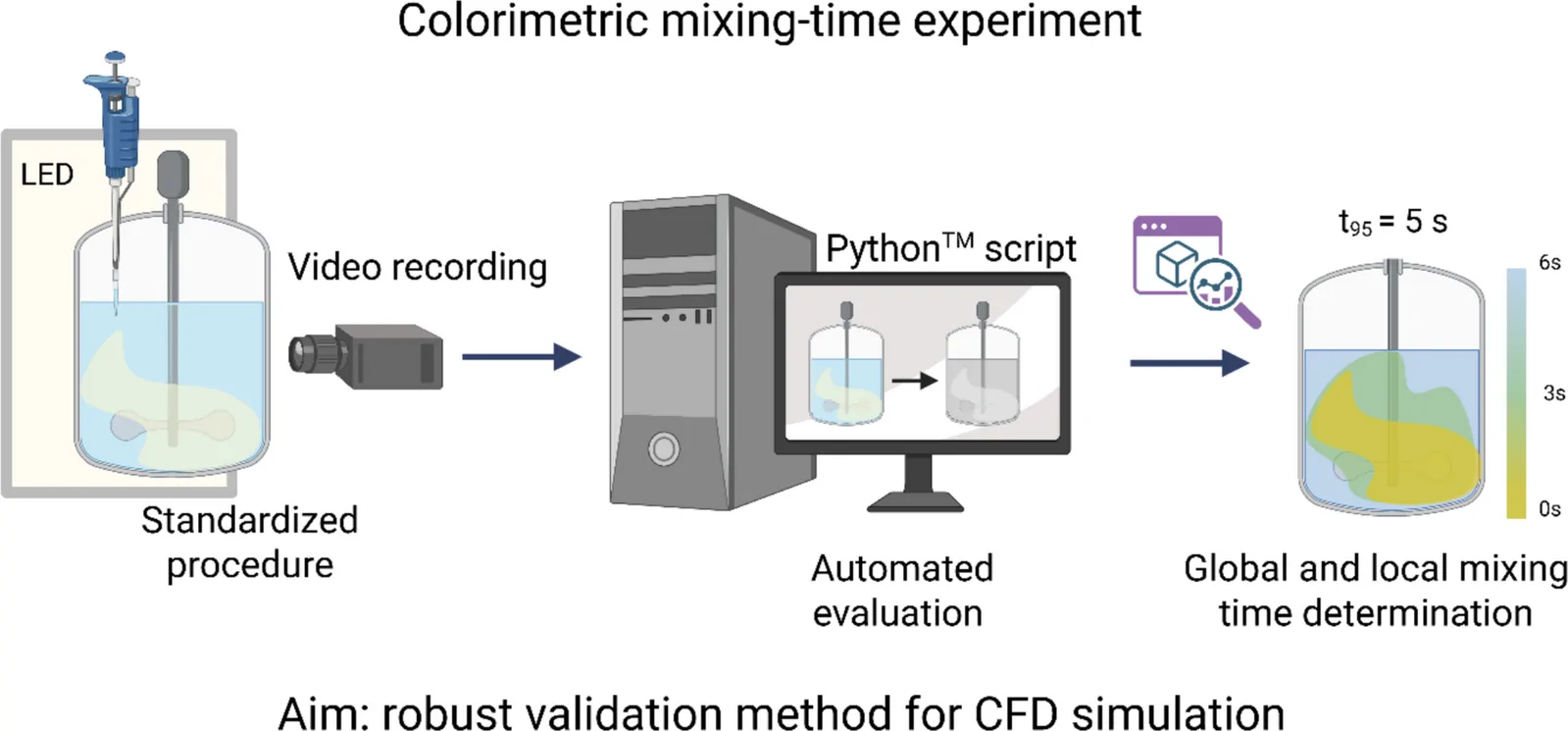

**Supplementary information:**

The online version contains supplementary material available at 10.1007/s00253-026-13941-8.

## Introduction

Mixing aims to homogenize multiple phases by equalizing concentrations and can be categorized into macro-, meso-, and micromixing. Macromixing is associated with large-scale flow above the Kolmogorov scale, mesomixing with smaller local flows, and micromixing with molecular diffusion and disintegration of turbulent eddies (Mao and Yang 2017; Nagata 1975). At all scales, mixing depends on the geometry of the apparatus and the operating conditions. Efficient fluid mixing is critical for biotechnological processes, particularly in bioreactors, where mixture homogeneity significantly influences reaction kinetics, microbial growth, and product yield (Bylund et al. 1998; Gaugler et al. 2023; Gumery et al. 2009). The need for effective mixing also extends beyond the preceding processing steps. It is also essential for downstream processing steps, such as crystallization, ultrafiltration/diafiltration (UF/DF), and formulation, where uniform distribution is required to ensure consistent product quality and process efficiency (Fournier et al. 1996; Gikanga et al. 2015).

The dimensionless local concentration, *θ*(*x*,*t*), describes the mixing state at position *x* and time *t* (Eq. 1). The local mixing time, *t*_mix_(*x*), is defined as the time at which the deviation of θ(x,t) from the steady-state value, *θ*_∞_(*x*) falls within a specified tolerance *ε*, that is, |*θ*(*x*,*t*) − *θ*_∞_(*x*)|< *ε*. Commonly used tolerances are *ε* = 0.1, *ε* = 0.05, and *ε* = 0.01. A tolerance of *ε *= 0.01 is often impractical, as actual inhomogeneities become indistinguishable from measurement noise at this level. Conversely, a threshold of *ε* = 0.1 allows for deviations of up to 10%, which is considered insufficiently precise for robust process characterization. Consequently, *ε* = 0.05 was selected for the present study, as it provides a reasonable balance between ensuring adequate homogeneity and maintaining a practically attainable level of measurement accuracy.

1 $$\theta \left(x,t\right)=\frac{c(x,t)-c(0)}{c(\infty )-c(0)}$$

where θ(*x,t*) is the dimensionless local concentration at position* x* and time* t*,* c*(*x,*t) is the measured value (e.g., concentration, pH, or color) at position* x* and time* t*,* c*(*0*) is the measured value at the start of the experiment, and* c*(∞) is the steady-state final value.

The global mixing time corresponds to the maximum local mixing time within the entire reactor volume and is therefore determined by the regions with the slowest mixing behavior. However, determining the global mixing time is highly susceptible to stochastic noise, making the result strongly dependent on individual outliers rather than the overall system state. Consequently, the spatial average of *θ*(*x,t*) is often used instead. In this case, the average mixing time is defined as the time at which |*θ*(*t*) − *θ*_∞_|< *ε* is achieved. Nevertheless, both definitions generally yield comparable results in well-mixed systems.

Traditionally, mixing times are determined using either tracer-based methods with probes, such as conductivity measurements or colorimetric visual assessment techniques employing dyes such as bromothymol blue or iodine-starch systems (Kraume and Zehner 2001; Löffelholz et al. 2013). In tracer-based methods, a tracer substance such as an acid/base or salt solution is introduced into the bioreactor. The tracer signal is then detected using probes positioned within the system, such as pH or conductivity sensors. By monitoring tracer concentration changes over time at various reactor locations, researchers obtain quantitative data on mixing efficiency and dynamics. However, the effectiveness of tracer-based approaches strongly depends on probe placement, which can considerably impact the accuracy and representativeness of the measurements (Fitschen et al. 2021). In contrast, colorimetric methods provide comprehensive spatial coverage of the system. The operator visually monitors color changes in the reactor after the injection of a liquid tracer. Common decolorization methods use iodine and thiosulfate with starch addition or pH indicators combined with acid-base reactions (e.g., phenolphthalein or bromothymol blue). The technique is nonintrusive and enables not only the determination of mixing time but also the qualitative visualization of flow patterns (Ascanio 2015).

However, colorimetric methods introduce operator dependency, which increases variability and may bias the results. Recent advancements in automation and imaging technologies provide opportunities to enhance the precision and reliability of these measurements. Integrating colorimetric methods with image analysis provides several distinct advantages, including comprehensive spatial coverage of the vessel and high spatial resolution. These features enable the determination of local mixing times (Bartczak and Pilarek 2024; Cabaret et al. 2007; Chhabra et al. 2007; Fitschen et al. 2021). Nevertheless, several challenges remain, including the need for vessel transparency and the occurrence of light reflections from internal structures, which can disrupt the analysis and introduce artifacts into the data. These factors require careful calibration, thoughtful experimental design, and implementation of strategies that minimize their impact and improve methodological reliability.

In addition to the methods discussed above, mixing in vessels can be evaluated by several alternative techniques. Electrical resistance tomography (ERT) visualizes the distribution of electrical conductivity in cross-sectional images (Ascanio 2015). These images can be converted into two-dimensional tomograms at different reactor heights and subsequently reconstructed into three-dimensional flow fields. Although the method can be applied to non-transparent systems, it is expensive and requires time-consuming setup procedures. Planar laser-induced fluorescence technique (pLIF) represents another advanced but expensive technique that uses lasers to visualize a fluorescent tracer (Distelhoff 1997). This tracer emits fluorescence that is tracked using photodiodes. Advantages of these direct measurement methods are high temporal and spatial resolution, nonintrusive operation, and the ability to measure different flow field variables, such as concentration, density, and temperature (Ascanio 2015). Thermography using thermochromic liquid crystals constitutes another approach for visualizing flow fields. The crystals rapidly and reversibly change color in response to temperature variations, thereby enabling the determination of mixing times and dynamic temperature changes (Lee and Yianneskis 1997). However, this method requires sophisticated and time-consuming data processing (Ascanio 2015).

Computational fluid dynamics (CFD) simulations have long been used to characterize mixing performance in stirred tank reactors (Mittal and Kikugawa 2021; Jenne and Reuss 1999; Patil et al. 2018; Sahu et al. 1999; Wutz et al. 2020), including models based on Reynolds-averaged Navier–Stokes (RANS) equations. Advances in computer simulation technology increasingly enable accurate and computationally sophisticated CFD-based predictions of flow behavior using dynamic approaches such as large-eddy simulation (LES) (Fitschen et al. 2021; Hartmann et al. 2006; Kuschel et al. 2021). CFD simulations enable the evaluation of various design alternatives and assess their effects on flow behavior during mixing processes in bioreactors (Ameur 2015; Ammar et al. 2011; Liu et al. 2024). Consequently, CFD can support in-silico optimization and reduce the need for time-consuming and costly optimization studies (Thomas et al. 2021). It is, therefore, necessary to compare the results of CFD simulations with real experimental data to determine the validity of the selected settings (Babuska and Oden 2004; ASME 2018). A CFD model can only be assumed to represent the real application accurately if it is validated using high-fidelity, high-resolution experimental data, which conventional probe-based methods cannot provide. To achieve meaningful validation, the experimental procedure and post-processing should resemble each other as closely as possible (Hartmann et al. 2006). Once validated, the CFD model can support optimization studies and moderate design modifications without requiring extensive experimentation.

Experimental investigations, including mixing-time experiments, are inherently subject to statistical variation and operator-dependent deviations. If experimental datasets with excessive variability are used for CFD validation, incorrect conclusions regarding simulation quality may result. Reliable interpretation, therefore, requires sufficiently reproducible experiments. Based on this consideration, the aim of this work was to develop a standard operating procedure (SOP) for mixing-time experiments combined with person-independent data evaluation. This approach seeks to minimize experimental variability and thereby improve CFD validation. Additionally, this work aims to provide a publicly available analysis tool that can be integrated into an SOP to support more accurate and reliable evaluation of mixing processes and ultimately improve bioreactor design and operations. Therefore, this study presents an automated methodology for determining local mixing times in stirred bioreactors. The approach uses a bromothymol blue color reaction induced by a pH shift, monitored through high-resolution video capture and analyzed using a custom-developed Python-based software tool. Although colorimetric methodologies are well established, this methodology extends these approaches by integrating automated analysis for determining local mixing times at specific locations within the bioreactor. This resulting spatially resolved data clarify the time required for individual regions of the bioreactor to reach equilibrium concentration and therefore provide a more detailed understanding of fluid dynamics during the mixing process. The tool also provides visualization of different mixing indices that support a more robust comprehensive assessment of mixing behavior. Furthermore, to enhance reproducibility, inter-laboratory trials are conducted to support the development of a standardized operating procedure (SOP). The preliminary results of the inter-laboratory trials are presented, as the tests are still ongoing. 

## Material and methods

In this study, mixing time was assessed using a colorimetric method with the pH indicator bromothymol blue. The color change was recorded using a video camera. The evaluation of mixing time was automated through a Python script, based on grayscale values, which represented the color change of the indicator (see Determination of global and local mixing times section). The combination of the colorimetric method and video recording also enabled the determination of mixing times at specific locations within the vessel, referred to as local mixing times. The following sections describe the automated evaluation process in detail.

### Mixing-time experiment

Mixing time was determined through optical measurement of the colorimetric pH shift of bromothymol blue from blue (alkaline) to yellow (acidic). The experiment was recorded using a Nikon D7500 camera (Nikon Corp.) equipped with a Zeiss Milvus 2.0/50 M ZF.2 lens (Carl Zeiss AG). The camera was positioned 800 mm to the center of the stirrer shaft, and the focal plane was aligned with the stirrer shaft. Videos were recorded with a spatial resolution of 1920 × 1080 px^2^ and a temporal resolution of 60 Hz. The bioreactor was positioned in front of an LED panel (Panel Value 1200 × 600 TPB 65 W 4000 K WT; Ledvance GmbH) equipped with a constant current supply controlled by an Arduino® Due microcontroller (Arduino s.r.l.) to prevent backlight flickering. This setup provided uniform illumination and ensured accurate color representation. Additionally, proper illumination reduced noise in darker regions and minimized interference from external light sources near the observed device (Bartczak and Pilarek 2024). The Arduino® Due microcontroller also synchronized activation of the backlight with the start of the mixing-time experiment. To define the aperture and exposure settings of the camera, the pH was adjusted to the neutral point under the corresponding backlight conditions. If necessary, the camera settings were readjusted under both acidic and alkaline conditions to enable sufficient illumination of the bioreactor. It was particularly important to avoid signal clipping at both the upper and lower intensity limits under all experimental conditions, including both the colored and decolored states of bromothymol blue. Exposure time, aperture, and ISO sensitivity were adjusted so that none of the states were overexposed or underexposed. This adjustment was achieved using the camera’s live histogram, which should display a balanced distribution of light and color values (Supplementary Information (SI) Fig. S1). Once established, these values remained unchanged throughout the experiment. Camera settings were kept constant within each experimental setup but could vary between laboratories because of differences in local lighting conditions.

**Fig. 1:**
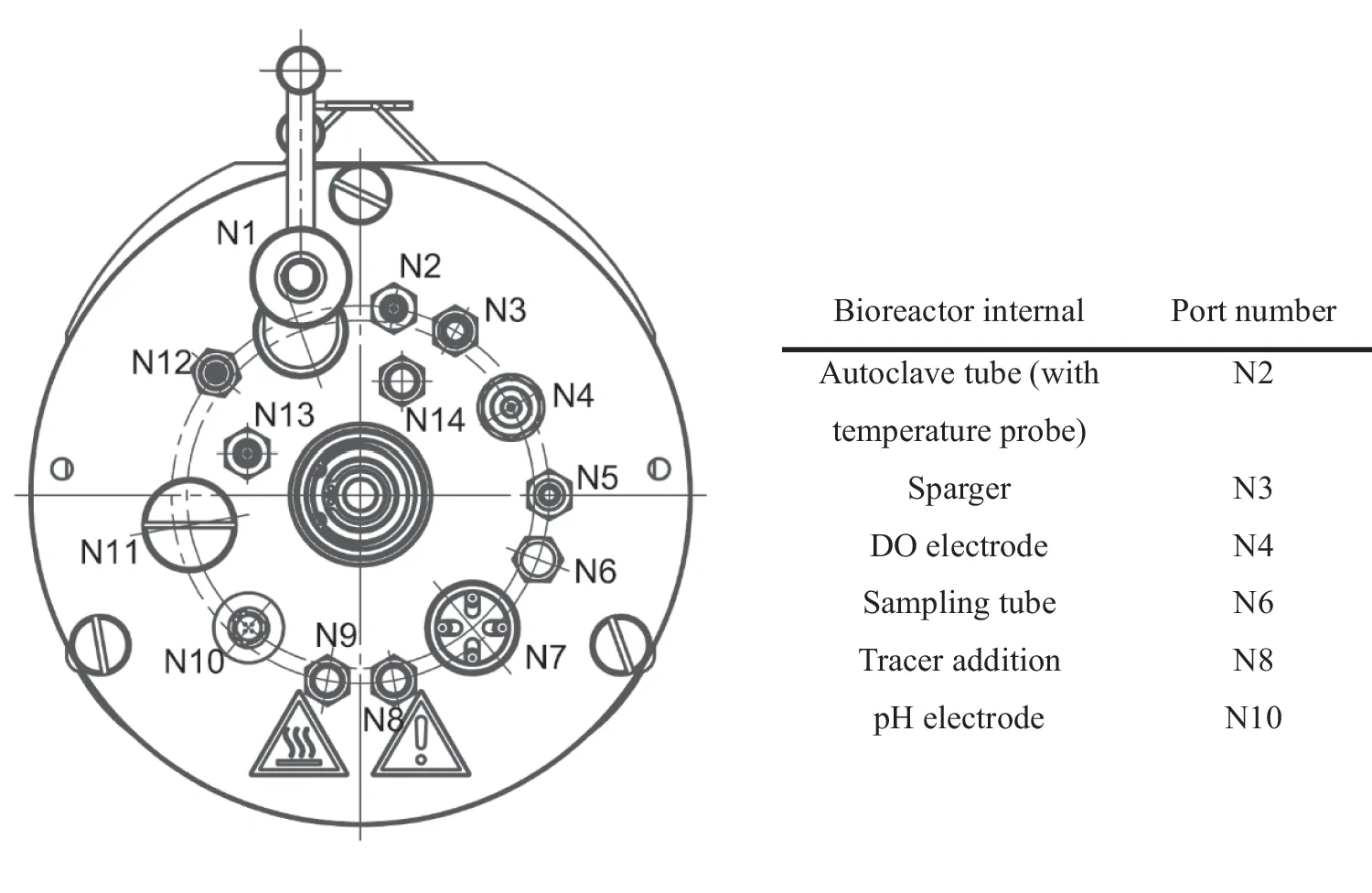
Univessel® 2 L DW (double-walled) glass bioreactor (Sartorius Stedim Biotech GmbH) lid configuration and port assignment

During the initial development of the methodology, glass vessels were used because their superior transparency and temperature-control capabilities enabled more accurate observations. The transfer of the method to single-use bioreactors and associated methodological challenges are described in Barth et al. (2026). The experiments were performed using the Univessel® 2 L DW (double-walled) glass bioreactor (Sartorius Stedim Biotech GmbH). This bioreactor provided good visibility, which was essential determining local mixing times. To ensure comparability among participants in the round-robin study, the participants agreed on a standardized reactor configuration, including lid port assignments (Fig. 1). The stirrer configuration consisted of a two-stage stirrer equipped with three-bladed segment impellers with a blade angle of 30° and a diameter of 54 mm (*D*_impeller_/*D*_vessel_ = 0.42). The impellers were installed at defined positions: the first impeller at a height of 24.5 mm and the second at 94.7 mm, both measured from the lower end of the stirrer shaft. The bioreactor internals included a dissolved oxygen electrode OxyFerm® DO (Hamilton Germany GmbH), a pH electrode EasyFerm® (Hamilton Germany GmbH), a ring sparger, a temperature probe (211 mm), and a straight dip tube.

The bioreactor was filled with 2 L deionized water, followed by the addition of 200 µL bromothymol blue solution (50 mmol/L in ethanol). The temperature was set to 25 °C and the desired stirring speed and aeration rate were set. The pH was adjusted dropwise to neutral using 4 M NaOH, using the pH-dependent color change of bromothymol blue as an indicator. Experiments were conducted in triplicate for each combination of stirring speed (50 rpm, 100 rpm, 212 rpm, 350 rpm and 424 rpm) and aeration rate (0 sLpm, 0.55 sLpm, and 1 sLpm). Subsequently, 45 µL NaOH (2 M) was added to the bioreactor. After 120 s, the backlight was switched off, and video recording was initiated. The following steps were then performed simultaneously: using a mechanical pipette (Eppendorf Research® from Eppendorf Vertrieb Deutschland GmbH, Finnpipette F1 from Thermo Fisher Scientific Inc., or a comparable pipette), 180 µL of a 2 M HCl solution was added at the exact moment the backlight was switched on. The pipette tip was immersed approximately 2 cm below the liquid surface at a 90° angle relative to the bioreactor lid through port N8, corresponding to a radial position of r = 51 mm from the center of the stirrer shaft. The port was aligned along the axis between the camera and stirrer shaft. Immediately after addition, the pipette tip was removed from the liquid. Video recording ended 30 s after complete yellow colorization had been achieved.

### Determination of global and local mixing times

To quantitatively determine mixing times, the Python-based script “MixingTimeAnalysis” was used to evaluate the recorded videos. The original evaluation was based on a MATLAB App Designer script developed by J. Fitschen et al. (2021) using several MATLAB toolboxes. However, since MATLAB is commercial software, the complete program was rebuilt and implemented in Python. This transition makes the script freely accessible to all users (For more information, see the “Statements and Declarations” section). Moreover, several new functions were integrated, including the definition of different thresholds, indices for quantifying homogeneity and coefficient-of-variation (CV), and the selective exclusion of image regions. The video analysis workflow implemented in the Python script is described below.

#### Setup of the analysis

Executing the Python file launched a setup window (SI Fig. S2). In this interface, users could select and load a video and adjust various settings. For instance, users could load an existing mask, select a region of interest (ROI) (see Definition of the analysis area section), or create an interactive map. Additional advanced settings, such as thresholds or smoothing parameters, could also be adjusted (SI Fig. S3).

#### Selection of the time range for analysis

In the next step, the start and end frames were defined and displayed through a graphical user interface with sliders and a preview function (SI Fig. S4). This procedure enabled precise selection of the relevant mixing-time interval while excluding irrelevant pre- or post-processing phases that could still be present in the video recordings.

#### Definition of the analysis area

To define the analysis area, either a previously generated mask was loaded in the setup window (see Setup of the analysis section) or a new mask was created. In the latter case, an additional window opened to enable mask selection (SI Fig. S5). The mask was initially generated by selecting a reference color within the start frame using the mouse, typically at a point located in the center of the colored solution. The software then selected all pixels with similar color values within a predefined interval. This procedure isolated the vessel contents with excluding regions outside the specified color range from further calculations. If the automatic selection did not fully cover the reactor contents, the tolerance interval could be adjusted using a slider to include or exclude additional pixels from the mask. In a second step, areas surrounding probes or stirrers could be excluded (SI Fig. S6). This step was particularly important for the impellers, as their orientation depended on the selected frame. After masking, only the unmasked area was evaluated. Therefore, a third window displayed the selected analysis area for user confirmation (SI Fig. S7). Once approved, the mask was saved, enabling reuse during evaluation of multiple video series. Furthermore, an ROI could be defined interactively (SI Fig. S8). Appropriate ROI selection enabled the identification of local differences in mixing times within the analyzed area. Once the analysis region had been defined, the actual video evaluation could begin.

#### Grayscale conversion

For grayscale conversion, each video frame was processed individually. Pixels in the digital image containing information in the red, green, and blue channels were converted into grayscale values using weighted contributions from the individual color channels. Conversion was performed using the OpenCV library function BGR2GRAY (Khan 2023). This conversion generated an image containing only information about light intensity, which was suitable for representing the color change of the indicator. The purpose was not to determine concentrations within the bioreactor, but rather to quantify indicator color gradient. The grayscale value *G*_pix_ was calculated for each pixel in every frame, resulting in grayscale-value matrices for all video frames (Eq. 2).

2 $${G}_{\mathrm{p}\mathrm{i}\mathrm{x}}=0.299\cdot r+0.587\cdot g+0.114\cdot b$$

where *r* is the red channel, *g* is the green channel, and *b* is the blue channel (Khan 2023).

#### Calculation of global mixing dynamics

For each video frame within the previously selected time interval (see Selection of the time range for analysis section), the mean intensity value *I(t)* within the unmasked area was calculated using the grayscale matrices *G*_pix_ according to the “Grayscale conversion” section. Normalization of the mixing signal was based on the initial state *I*_0_ and a reference steady state *I*_ref_. Specifically, 0% corresponded to the initial mean intensity, while 100% was defined as the mean intensity averaged over a final reference interval at the end of the experiment. The global normalized mixing index *S*(*t*) was calculated according to Eq. 3.

3 $${S}\left({t}\right)=\frac{{I}\left({t}\right)-{I}_{0}}{{I}_{ref}-{I}_{0}}\cdot 100$$

To reduce sensitivity to measurement noise and short-term fluctuations, the mixing curves were smoothed using a temporal moving average prior to further analysis (Eq. 4). The implementation used a finite moving-average window determined by the selected smoothing duration *n*, the frame duration Δt, and the number of frames *N*_f_ included in the smoothing procedure.

4 $$\overline{\mathrm{S}}\left(\mathrm{t}\right)=\frac{1}{{\mathrm{N}}_{\mathrm{f}}}\sum\nolimits_{\mathrm{k}=-\mathrm{n}}^{\mathrm{n}}\mathrm{S}\left(\mathrm{t}+\mathrm{k}\Delta \mathrm{t}\right)$$

The mixing time *t*_mix_ was determined using a final-band criterion. In this approach, mixing time was defined as the final entry into a tolerance band around the steady-state value, evaluated backward from the end of the signal (Eq. 5). This criterion ensured that all subsequent values remained within the specified band and excluded transient excursions effectively.

5 $${t}_{\mathrm{m}\mathrm{i}\mathrm{x}}=\mathrm{m}\mathrm{i}\mathrm{n}\left\{\mathrm{t}\hspace{0.33em}|\hspace{0.33em}\overline{\mathrm{S}}\left(\uptau \right)\in \left[\left(1-\upvarepsilon \right),\hspace{0.17em}\left(1+\upvarepsilon \right)\right]\hspace{0.33em}\forall\uptau \ge \mathrm{t}\right\}$$

For *t*_95_, the tolerance band corresponds to 1 ± 0.05.

#### Mixing metrics

In addition to the normalized mean intensity, several alternative mixing indices were evaluated, including the endpoint deviation index, the homogeneity index, and the CV reduction index.

The endpoint deviation *E*(*t*) quantifies the spatially averaged absolute deviation from the final reference image within the unmasked area Ω (Eq. 6).

6 $${E}\left({t}\right)=\frac{1}{\left|{\Omega}\right|}{\sum}_{{x}\in{\Omega}}\left|{I}\left({x},{t}\right)-{I}_{{ref}}({x})\right|$$

The endpoint deviations *E*(*t*) were normalized to the deviation of the first frame *E*_0_, resulting in the asymptotic endpoint deviation index *M*_e_(*t*) (Eq. 7).

7 $${M}_{e}\left(t\right)=\left(1-\frac{E\left(t\right)}{{E}_{0}}\right)\cdot 100$$

The homogeneity index *M*_h_(*t*) was calculated using the relative standard deviation (Eq. 8) according to Nagata (1975). This index indicates whether the system is homogenously distributed.

8 $${M}_{h}\left(t\right)=\left(1-\frac{\sigma \left(t\right)}{{\sigma}_{0}}\right)\cdot 100$$

The standard deviation *σ*(*t*) was calculated from the spatially resolved intensities *I*(*i*,*t*) according to Eq. 9, where *N*_p_ denotes the number of pixels within the unmasked area Ω and µ represents the mean pixel intensity within Ω.

9 $$\sigma (t)=\sqrt{\frac{1}{{N}_{p}}{\sum}_{i=1}^{{N}_{p}}{\left(\mathrm{I}(i,t)-\mu (t)\right)}^{2}}$$

The CV reduction index *M*_CV_(*t*) normalized the spatial variance by the mean intensity and was therefore sensitive to relative fluctuations (Eqs. 10 and 11).

10 $$CV\left(t\right)=\frac{\sigma \left(t\right)}{\left|I\left(t\right)\right|}$$

11 $${M}_{CV}\left(t\right)=\left(1-\frac{CV\left(t\right)}{C{V}_{0}}\right)\cdot 100$$

#### Spatially resolved mixing behavior

Local mixing times ${t}_{\mathrm{m}\mathrm{i}\mathrm{x}}\left(x\right)$ were defined on a pixel-wise basis. For each pixel, the mixing state was evaluated relative to the final reference image. A pixel was considered mixed once its absolute deviation from the final state fell below a specified fraction of its initial deviation. For *ε* = 0.05, the remaining local endpoint deviation had to be less than or equal to 0.05 of the initial local deviation. Equation 12 was derived from Eq. 1, but intensity values were used instead of concentrations according to the selected colorimetric mixing-time method.

12 $${t}_{\mathrm{m}\mathrm{i}\mathrm{x}}\left(x\right)=\mathrm{m}\mathrm{i}\mathrm{n}\left\{t\hspace{0.33em}|\hspace{0.33em}\left|I\left(x,t\right)-{I}_{\mathrm{r}\mathrm{e}\mathrm{f}}\left(x\right)\right|\le \left(1-\upvarepsilon \right)\cdot \left|I\left(x,0\right)-{I}_{\mathrm{r}\mathrm{e}\mathrm{f}}\left(x\right)\right|\right\}$$

The unmixed fraction *f*_unmixed_ represents the proportion of pixels *N*_p_ that did not satisfy the local mixing criterion within the observation period. It was calculated according to Eq. 13.

13 $${f}_{\mathrm{u}\mathrm{n}\mathrm{m}\mathrm{i}\mathrm{x}\mathrm{e}\mathrm{d}}=\frac{{N}_{\mathrm{p},\mathrm{u}\mathrm{n}\mathrm{m}\mathrm{i}\mathrm{x}\mathrm{e}\mathrm{d}}}{{N}_{\mathrm{p},\mathrm{t}\mathrm{o}\mathrm{t}\mathrm{a}\mathrm{l}}}$$

To quantify spatial heterogeneity in a robust manner, percentile-based mixing times *p*_q_ were evaluated (Eq. 14). Here, ${Q}_{q}$ denotes the *q*^th^ percentile of the distribution of pixel-wise local mixing times. In particular, ${p}_{95}$ represents the time required for 95% of the unmasked area Ω to reach the mixed state.

14 $${p}_{q}={Q}_{q}\left(\left\{{t}_{\mathrm{m}\mathrm{i}\mathrm{x}}\left(x\right)\mid x\in \Omega \right\}\right)$$

#### Visualization and presentation of results

The overview figure (SI Fig. S10) contains plots of different global mixing metrics, including the mean intensity index $\overline{S }(t)$, endpoint deviation index ${M}_{e}\left(t\right)$, homogeneity index ${M}_{h}\left(t\right),$ and CV reduction index ${M}_{CV}\left(t\right)$. The time points “*t*_95_ final band mean” (calculated from $\overline{S }(t)$ according to the “Calculation of global mixing dynamics” section) and “*t*_95_ final band endpoint” (calculated from *M*_e_*(t)* according to the “Mixing metrics” section) are displayed as vertical markers. Additionally, the spatial standard deviation $\sigma \left(t\right)$ is plotted against time on the right side of the overview figure, whereas the non-normalized raw intensity values are shown in the left center. The local mixing times ${t}_{\mathrm{m}\mathrm{i}\mathrm{x}}\left(x\right)$ are visualized as a two-dimensional color-coded chart, making spatial differences in mixing behavior readily apparent. A histogram illustrating the frequency distribution of local mixing times is displayed in the lower-right corner.

If selected during the initial setup step (see Setup of the analysis section), the window “interactive global mixing curves” opens (SI Fig. S11). In this interface, users can select time points with the cursor to display the corresponding values of the mean intensity index, endpoint deviation index, homogeneity index, and CV reduction index.

Optionally, intermediate mixing states can be exported as two-dimensional color-coded images to visualize the temporal progression of homogenization. By default, 10 intermediate images are generated, although users can modify this number through the advanced settings (see Setup of the analysis section and SI Fig. S3). The progress of the evaluation is then shown in the command window (SI Fig. S9).

All results were automatically saved in a results folder with a timestamp. In addition to the mask file stored in *.npy format, the software generated color-coded maps, a combined overview figure, and a histogram of local mixing times. Furthermore, an *.xlsx file was generated containing video metadata (e.g., number of frames, frame rate, and duration), the calculated mixing times *t*_95_, the complete time series, and the resulting figures. The software also included overview sheets for mixing quality and initial instability incorporating a traffic-light system and user recommendations. For example, the mixing quality sheet displayed the endpoint *t*_95_ and local *p*_95_ values together with a comparison of these parameters. Deviations below 5% indicated good mixing quality, whereas deviations over 20% indicated poor mixing quality. The software additionally provided suggestions for identifying poorly mixed regions based on the two-dimensional color-coded maps.

Additionally, a *.docx file containing summarized data and guidance for interpretation was generated automatically. This procedure ensured structured and reproducible documentation of all analysis results.

## Results and discussion

**Fig. 2:**
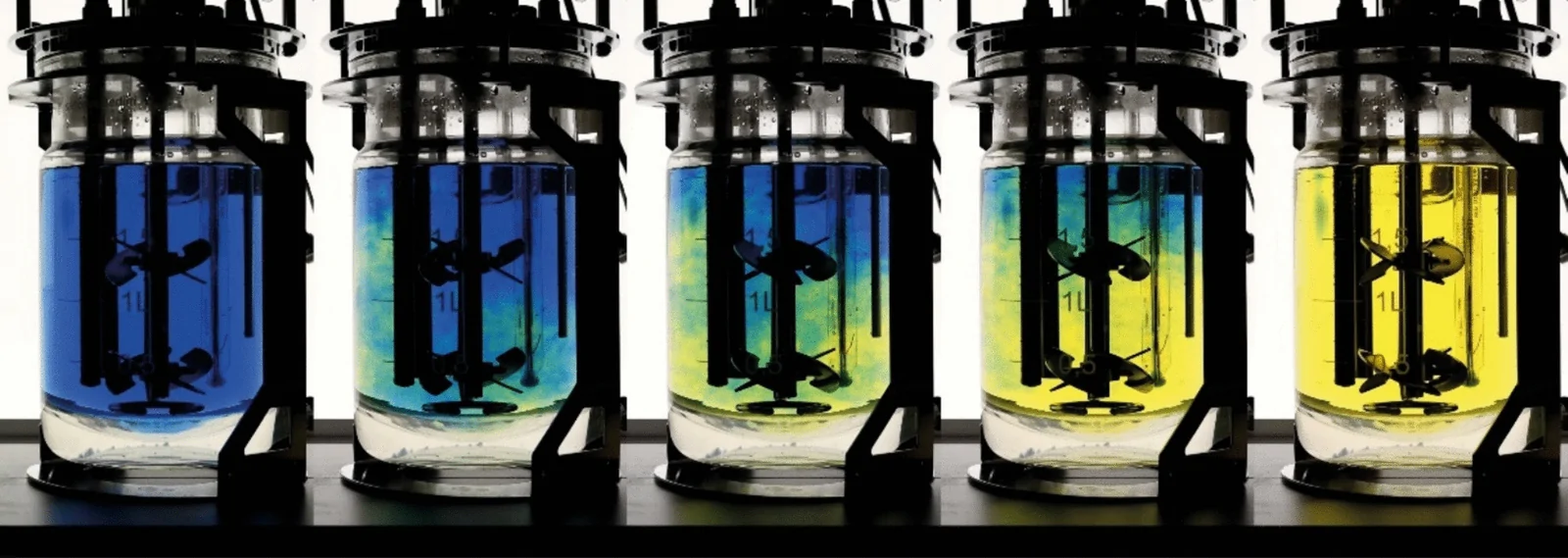
Different mixing states in the glass bioreactor. The left image displays the bioreactor with the alkaline solution prior to the addition of acid. Subsequent images from left to right illustrate the mixing progress at various time points, concluding with the fully mixed bioreactor on the far right

The optical measurements described in the “Mixing-time experiment” section were successfully performed in the glass bioreactor used in this study. Despite the double-walled reactor design, the interior of the bioreactor remained sufficiently visible for analysis (Fig. 2).

### Influence of masking

To evaluate the influence of masking, different mask configurations were compared. Selecting the mask using the color-picker function in the start frame before grayscale conversion enabled the user to isolate only the region inside the bioreactor. If the entire image was selected for analysis (SI Fig. S12), regions outside the bioreactor also appeared in the two-dimensional local mixing-time map. In this case, artifacts such as reflections could be included in the evaluation. If these reflections correlated with the color change inside the bioreactor, they could directly affect the calculated mixing indices because larger areas would require additional time to reach *t*_95_.

**Fig. 3:**
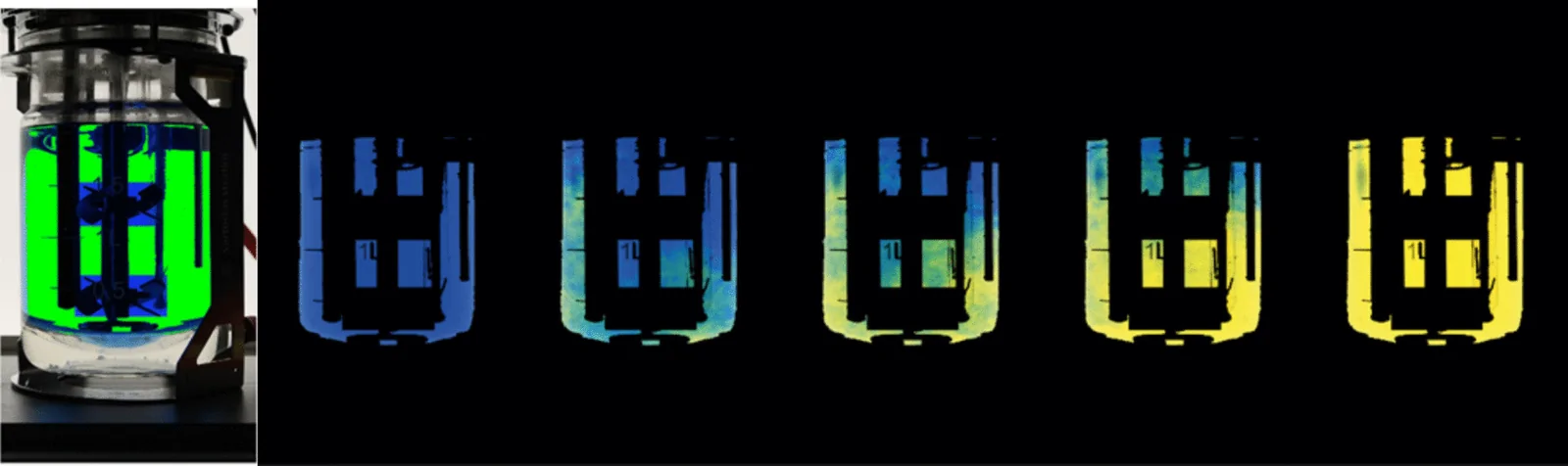
Different mixing states in the glass bioreactor after masking via the selection in the Python tool. The unmasked area with excluded stirrers is highlighted in green on the left side of the image. The second image from the left displays the unmasked region of the bioreactor with the alkaline solution prior to the addition of acid. Subsequent images from left to right illustrate the mixing progress at various time points, concluding with the fully mixed bioreactor on the far right

If the color-picker function was used without any additional modifications or exclusions, the local heatmap appeared to represent only the bioreactor region adequately. However, the overview window, particularly the spatial standard deviation plot, showed pronounced fluctuations (SI Fig. S13). Closer examination of the intermediate-state images (SI Fig. S14) revealed that these fluctuations originated from light reflections on the stainless-steel stirrers combined with the backlighting. Since impeller orientation depended on the selected frame (Fig. 2 and SI Fig. S14), stirrer movement caused periodic fluctuations and introduced signal noise. To minimize this effect, the stirrers had to be excluded from the analysis area during masking (Fig. 3). This adjustment substantially reduced the signal noise (SI Fig. S15). To ensure that slow mixed regions were not overlooked, the analyzed area should nevertheless include the entire bioreactor volume, including the upper and lower regions.

**Fig. 4:**
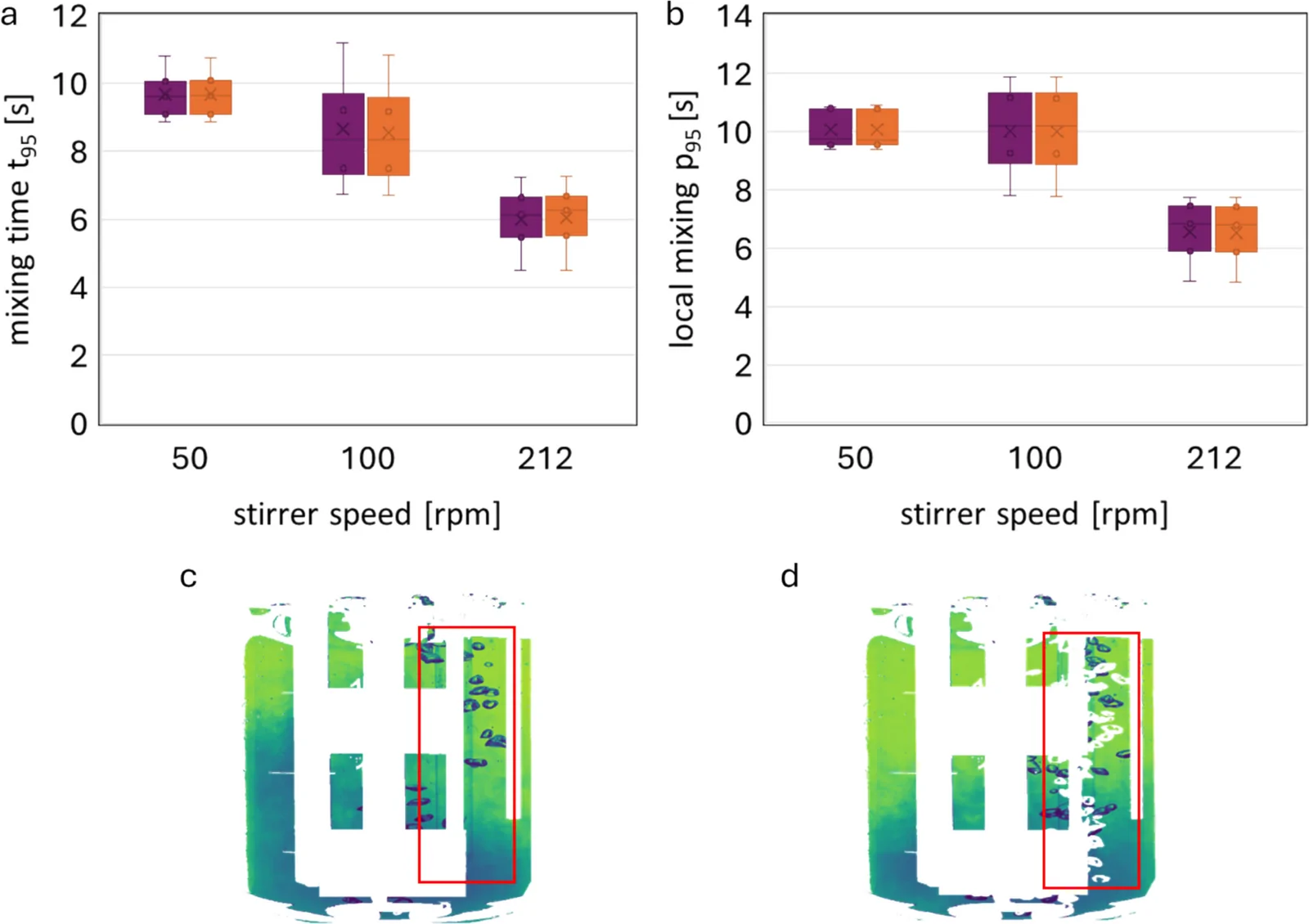
Comparison of masks for aerated experiments. Results for the mean t_95_ values (**a**) and local p_95_ values (**b**) at different stirring speeds are shown. Violet data correspond to unmasked areas without bubbles, whereas orange data correspond to unmasked area including bubbles. The images (**c** and **d**) show intermediate mixing states using different masks for one representative video evaluation at *t* = 4 s. The mask in the left image (**c**) does not include bubbles, whereas the mask in the right image (**d**) includes them, thereby reducing the unmasked area. Within the unmasked area, the underlying bubble shapes of the mask are highlighted in white, whereas the actual physical bubbles in the video frame are colored blue. Red rectangles highlight regions where visible differences between **c** and **d** arise due to the presence of rising bubbles

In aerated bioreactors, bubbles could theoretically be considered fluctuating regions. Nevertheless, masking the entire bubble-containing region is not recommended because large portions of the bioreactor volume would then be excluded. At present, the color-picker approach does not allow selective exclusion of bubbles visible in the start frame. One possible solution would involve an additional preprocessing step during mask generation that includes manually defined inclusion areas. Another option would be to record an additional video without aeration while maintaining the same camera position. However, the results showed that defining an unmasked area completely free of bubbles in the start frame was not necessary. Comparison of evaluations performed with and without visible bubbles in the unmasked area yielded similar values for both the mean *t*_95_ values and the local *p*_95_ values (Fig. 4a and b). Nevertheless, unmasked areas without bubbles improved the visualization of intermediate states of mixing (Fig. 4c and d) and in the two-dimensional color-coded local mixing-time maps.

To ensure that the results were not biased by manual definition of the analysis area, a repeatability test was performed. The start and end frames remained constant, whereas the mask was reselected 10 times for two representative videos. The mask was selected to ensure full coverage of the bioreactor volume while excluding stirrers and surrounding reflections. The resulting coefficients of variation of 0.1% for the mean *t*_95_ with aeration and 0.3% for the endpoint *t*_95_ without aeration confirmed that the impact of area selection was negligible and that the method provided highly consistent results.

### Influence of end-frame selection

The start frame was precisely defined through synchronization with the LED illumination. In contrast, end-frame selection remained user dependent and could therefore influence the evaluation results. Consequently, repeatability with different end-frame selections was investigated for both aerated and non-aerated experiments. For the aerated case, end frames were selected across a range of approximately 500 frames, whereas a range of approximately 2500 frames was used for the non-aerated case, both in increments of 50 frames. Additionally, single-frame increments were evaluated for the aerated video to assess the influence of bubbles. The repeatability of the evaluation using different end frames was confirmed by CVs of 0.4% for the mean *t*_95_ in non-aerated experiments and 1.1% for the aerated experiments when using 50-frame increments. For single-frame increments, a CV of 0.1% was obtained.

### Comparison and Interpretation of mixing indices

**Fig. 5:**
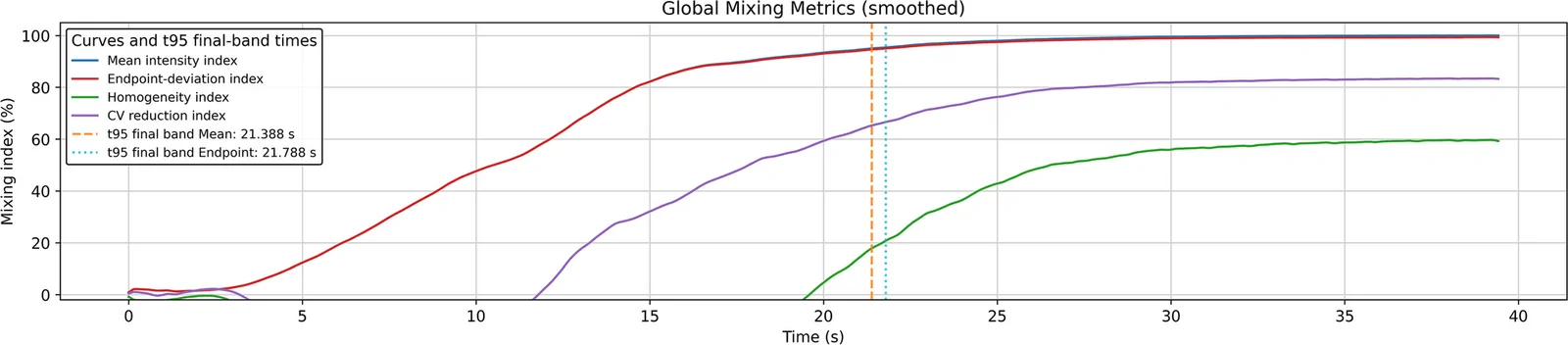
Upper selection of the overview window provided as output by the Python tool showing the global mixing metrics for one representative video evaluation of a video without aeration and with 50 rpm stirring speed. The graphic displays the mean intensity index (blue, covered by the red line), the endpoint deviation index (red), the homogeneity index (green), and the CV (coefficient of variation) reduction index (violet), together with the vertical markers for mean *t*_95_ (orange) and endpoint *t*_95_ (light blue). A complete representation of the overview window is provided in SI Fig. S10

After data processing in the Python script (according to the Setup of the analysis –Visualization and presentation of results sections), the first output was an overview combining different representations (SI Fig. S10). The upper part of the window contains a graph displaying the global normalized mixing index over time $\overline{S}$(t) (Fig. 5, blue line). The mixing curve exhibits characteristic asymptotic behavior and approaches a steady-state value as mixing progresses. In contrast to conventional threshold-based approaches, mixing time was determined using a final-band criterion. In this approach, mixing time is defined as the point at which the signal enters a tolerance band around the steady-state value when evaluated backward from the end of the measurement. The resulting global mixing times, particularly the mean *t*_95_, provide a robust estimate of overall mixing performance. The metric is highly resistant to outliers, particularly in well-mixed laboratory-scale bioreactors, and can therefore be used to compare reactors and their internal components. Compared with the classical first-threshold crossing, the final band criterion systematically yields slightly longer mixing times because it additionally requires temporal stability.

Several alternative mixing indices were also evaluated, including the endpoint deviation index, the homogeneity index, and the CV reduction index (see Mixing metrics section). While all mixing metrics exhibited similar qualitative trends, distinct differences arose in their sensitivity to spatial fluctuations. The endpoint deviation index *M*_e_*(t)* directly quantifies deviation from the final reference state and therefore showed smooth convergence behavior (Fig. 5, red line). Similar to the global normalized mixing index, this curve approached a steady-state value asymptomatically. In contrast, the homogeneity index *M*_h_(*t*) is based on the reduction of spatial standard deviation and therefore reflects the progressive elimination of concentration gradients (Fig. 5, green line). The CV reduction index *M*_CV_(*t*) normalizes the standard deviation by the mean intensity, making it particularly sensitive to relative fluctuations, especially in low-intensity regions (Fig. 5, violet line). Together, these indices provide a comprehensive and physically consistent description of the mixing process.

**Fig. 6:**
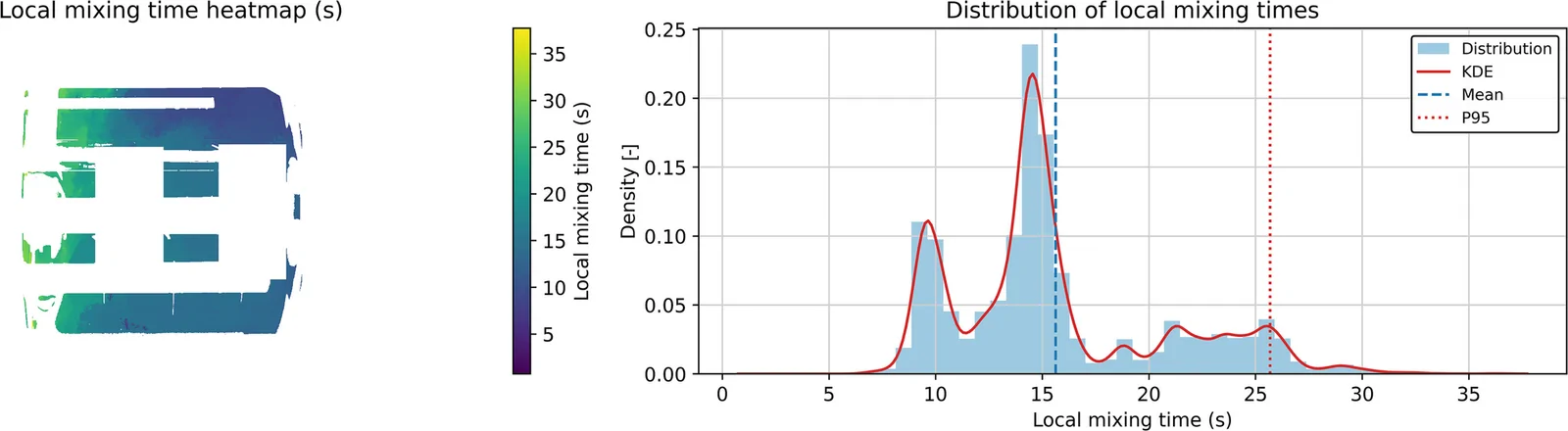
Lower section of the overview window provided as output by the Python tool for one representative video evaluation containing the two-dimensional color-coded local mixing-time map on the left side and the distribution of local mixing times (light blue) on the right side for a video without aeration and with 50 rpm stirring speed. The graph of the distribution of local mixing times further displays the kernel density estimator (KDE, red line) and two vertical markers showing the mean value (blue) and the p_95_ value (red). A complete representation of the overview window is provided in SI Fig. S10

One notable advantage of the decolorization method using bromothymol blue is its ability to provide visual information throughout the entire vessel volume. This capability facilitates the identification of regions in which mixing occurs most rapidly and regions in which mixing is slowest, making the assessment independent of probe placement. Furthermore, the method allows determination of mixing time at any specific location within the vessel. Consequently, the method enables a more detailed evaluation of mixing efficiency and provides insight into local variations in mixing performance. To support this evaluation, the lower section of the overview window (Fig. 6 and SI Fig. S10) contains two-dimensional color-coded charts showing the local mixing time *t*_95_(*x*) in seconds on the left side and the distribution of local mixing times on the right side. The distribution typically exhibits a right-skewed shape, indicating that although a large fraction of the domain mixes rapidly, a subset of regions requires considerably longer mixing times. Distribution width reflects the degree of spatial uniformity. A narrow distribution corresponds to homogeneous mixing, whereas a broad distribution indicates pronounced spatial variability. In the present case, the observed spread suggests coexistence of fast- and slow-mixing regions. The pronounced right-hand tail indicates the presence of slow-mixing zones, which are often associated with stagnant-flow regions or diffusion-limited transport processes (Fitschen 2022). In combination with the two-dimensional color-coded chart, the distribution allows conclusions regarding zone formation within the bioreactor. Depending on whether zone formation occurs, either a unimodal or multimodal distribution may occur. In the case of a unimodal distribution, the average mixing time is characteristic of the entire system. In contrast, a multimodal distribution may indicate the presence of zones with limited fluid exchange (Fitschen 2022).

To quantify spatial heterogeneity in a robust manner, percentile-based mixing times were evaluated. In particular, the *p*_95_ value represents the time required for 95% of the domain (unmasked area Ω) to reach the mixed state. This mixing metric is especially relevant from an engineering perspective, as it provides a conservative estimate of mixing performance by explicitly accounting for poorly mixed regions. When heterogeneities occur, comparison of the global *t*_95_ and local *p*_95_ values reveals a systematic discrepancy. This difference arises from the fundamentally different definitions of the two metrics. Global mixing times *t*_mix_ are derived from spatially averaged quantities, whereas local percentiles *p*_q_ are based on distributions of pixel-wise mixing times. Consequently, slow-mixing regions exert only limited influence on global metrics but strongly affect higher local percentiles. Therefore, the global *t*_95_ value can be used for comparison of different setups, as it is less sensitive to outliers. However, it shows limitations when heterogeneities or unmixed zones are present. In the Excel output file, the section “mixing quality” compares local and global data side by side and calculates the deviation between them. Based on this comparison, the software provides recommendations using a traffic-light system. If the local and global values are similar (green), the global values can be used without restriction for comparative purposes. If the values are further apart (yellow), consulting the local times is strongly recommended to identify heterogeneous regions. In cases of very large discrepancies, the software additionally recommends verifying the experimental setup for reflections, distortions, or wrong masking.

### Global mixing-time evaluation

**Fig. 7:**
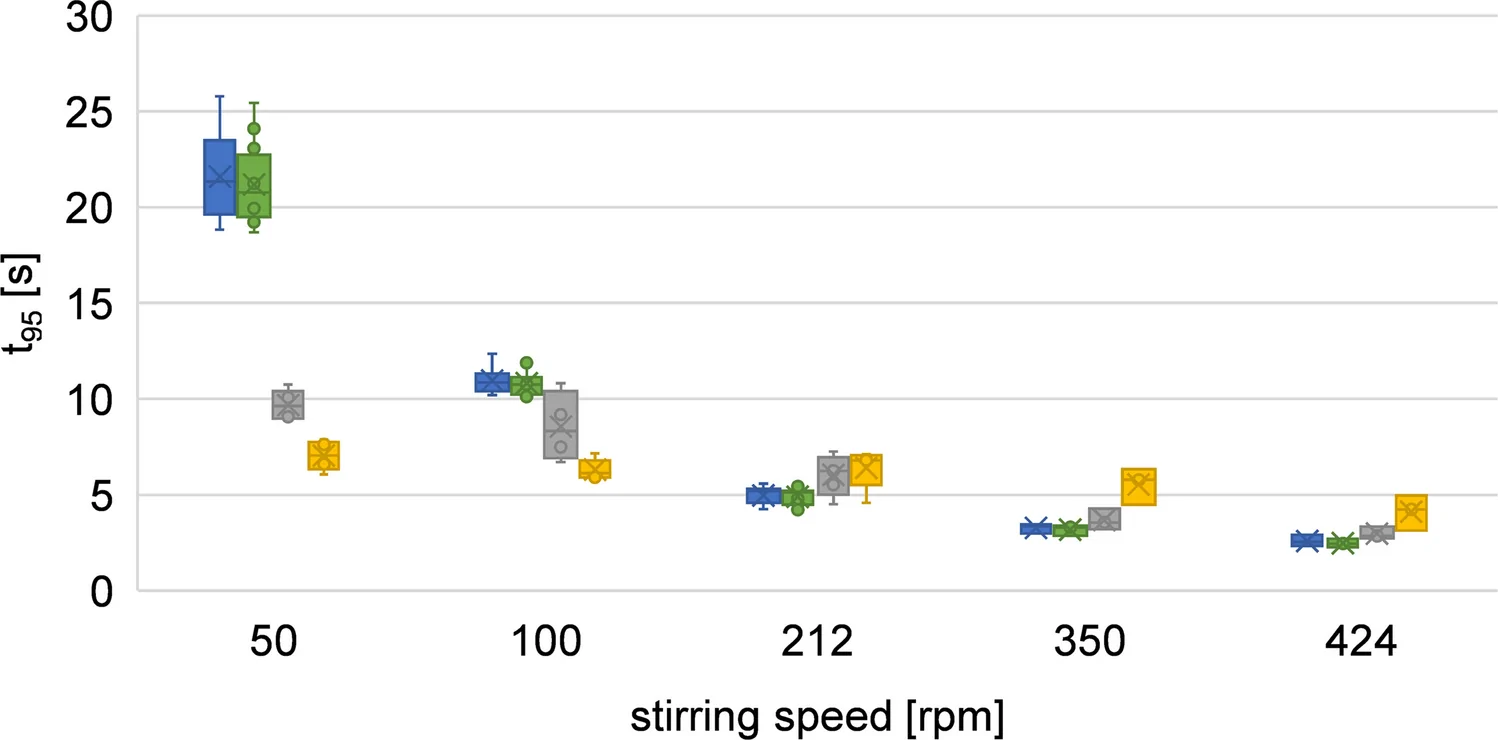
Global mixing times at different stirring speeds and aeration conditions in the Univessel® 2 L DW (double-walled) glass bioreactor: endpoint *t*_95_ without aeration (blue), mean *t*_95_ without aeration (green), mean *t*_95_ at 0.55 sLpm aeration (gray), mean *t*_95_ at 1 sLpm aeration (yellow)

To test the applicability of the Python script, mixing-time experiments were carried out at different stirring speeds and aeration rates, and the resulting data were compared. The endpoint deviation index *M*_e_(*t*) and the global normalized mixing index $\overline{S }(t)$ showed very similar behavior for the non-aerated experiments (SI Fig. S15), and the endpoint *t*_95_ and mean *t*_95_ values followed similar trends (Fig. 7, blue and green). As expected, the non-aerated experiments showed decreasing mixing times with increasing stirring speed. Therefore, both metrics can be used for comparison between different setups.

The videos recorded during aerated experiments could also be evaluated with the tool, although fluctuations caused by rising air bubbles introduced noticeable noise into the graphs. Since the gas bubbles were distributed unevenly throughout the volume, frame-to-frame comparison showed notable differences as the bubbles rose through the reactor. Consequently, the endpoint deviation index did not reach 100% in the aerated experiments. Therefore, the software could not calculate endpoint *t*_95_ values under aerated conditions. In these cases, only the mean *t*_95_ values from the global normalized mixing index were compared.

The mean global mixing time *t*_95_ decreased with increasing aeration at lower stirring speeds (50 rpm and 100 rpm), which can be explained by the transition from purely stirrer-driven flow to combined stirrer- and bubble-driven flow. At higher stirring speeds, however, this reduction was not observed (Fig. 7, green, gray, and yellow). Simultaneously, the air bubbles increased light reflections and thereby complicated signal evaluation, resulting in larger deviations compared to the non-aerated experiments. In the corresponding diagrams, *M*_e_(*t*) and $\overline{\mathrm{S}}\left(t\right)$ exhibited fluctuations, and the final-band determination of *t*_95_ became more sensitive to noise. Nevertheless, a consistent trend remained visible. As stirring speed increased, the reduction in mixing time caused by aeration became less pronounced. At the highest aeration rate combined with the highest stirring speed, aeration even resulted in slightly longer mixing times. These slightly longer or unchanged mixing times at high aeration rates and high stirring speeds may occur because large numbers of rising bubbles impede vertical fluid exchange, thereby reducing homogenization efficiency near the bottom of the bioreactor. This observation is consistent with the findings of Hadjiev et al. (2006), who also reported increased mixing times at higher aeration rates under high stirring speeds.

### Local mixing-time evaluation

The observations of longer mixing times at the bottom of the bioreactor under aerated conditions were confirmed by the local mixing-time evaluation. The two-dimensional color-coded maps and intermediate mixing-state images enabled direct visualization of such heterogeneities.

**Fig. 8:**
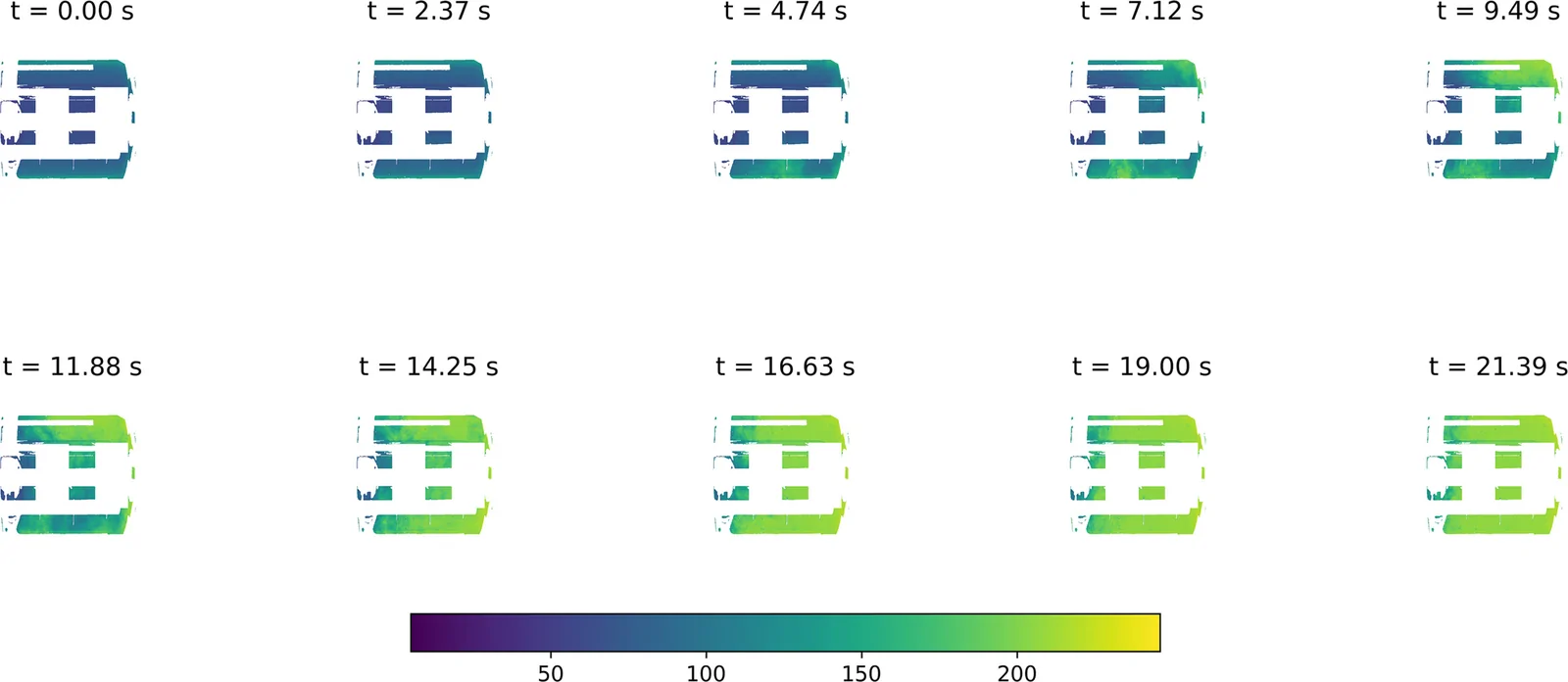
Ten partial states of the mixing in the bioreactor before the full homogeneity is reached for an experiment with 50 rpm and without aeration; the color bar shows a scale for the grayscale values with the alkaline parts of the liquid in blue and the acidic parts shown in yellow

**Fig. 9:**
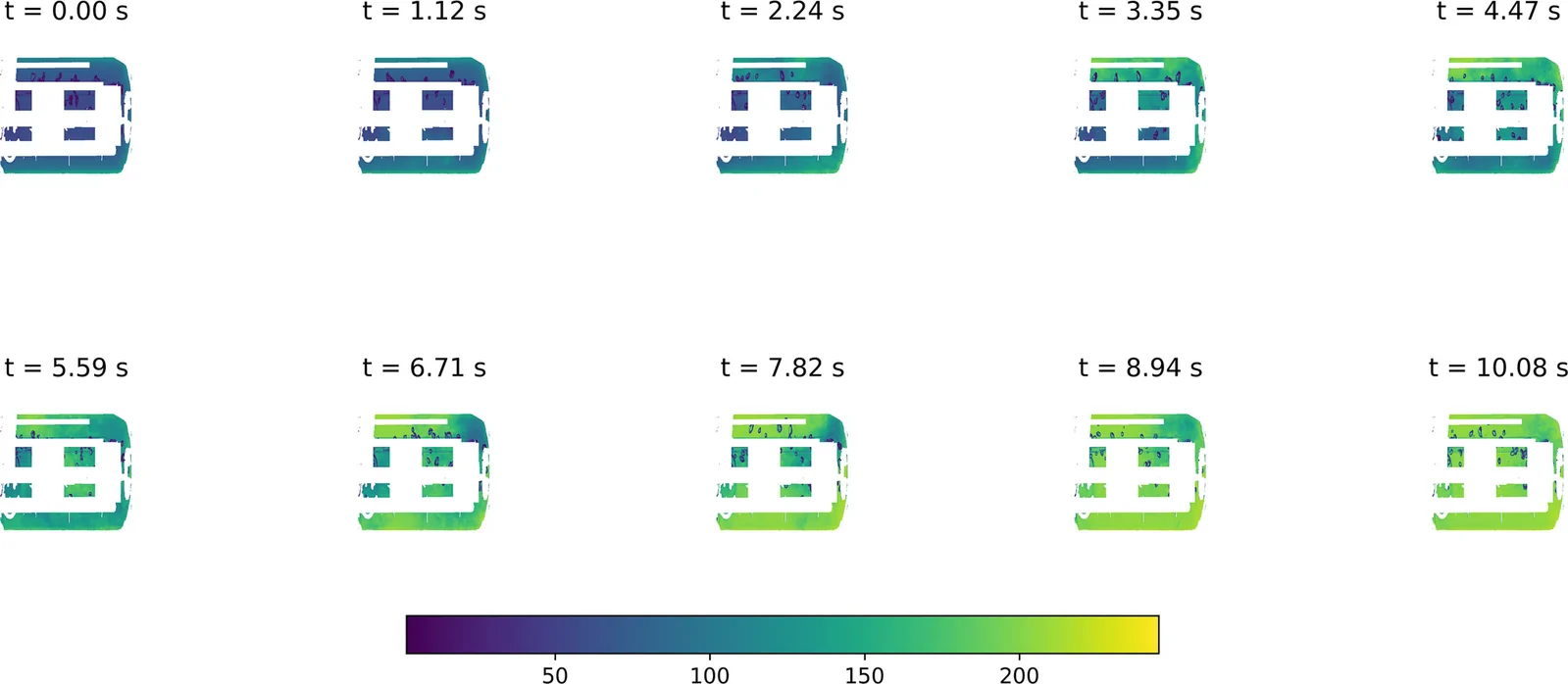
Ten partial states of the mixing in the bioreactor before the full homogeneity is reached for an experiment with 50 rpm and 0.55 sLpm aeration; the color bar shows a scale for the grayscale values with the alkaline parts of the liquid in blue and the acidic parts shown in yellow

As shown in Fig. 8, the slow-mixing region under non-aerated conditions was located in the upper part of the bioreactor. The injected acid is distributed to the area between the stirrers, then pushed downwards by the stirrer-driven flow. The acid is then rising along the walls towards the upper part of the bioreactor. In contrast, the aerated bioreactor shows the zone with the slowest mixing at the bottom (Fig. 9). This is due to the rising air bubbles (see Global mixing-time evaluation section), which make it difficult to replace the liquid under the ring sparger. In line with the results of the global mixing-time determination for the experiments with a rotational speed of 50 rpm, the interval two-dimensional color-coded charts (plots of the mixing state in the bioreactor at different times *t*) also show that an increase in aeration led to a reduction in the time (from 10.4 s to 7.5 s) required to achieve mean global mixing time.

**Fig. 10:**
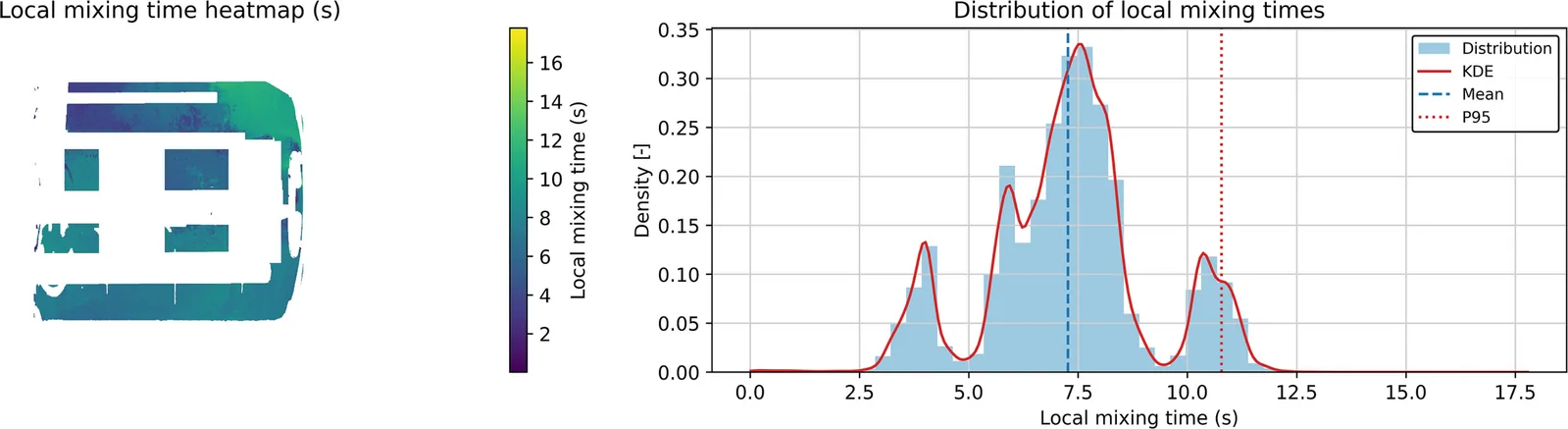
Two-dimensional color-coded diagram (local mixing-time heatmap) on the left side and the distribution of local mixing times (light blue) on the right side of a video of the experiment at 50 rpm with 0.55 sLpm aeration. The graph of the distribution of local mixing times further displays the kernel density estimator (KDE, red line) and two vertical markers showing the mean value (blue) and the *p*_95_ value (red). A complete representation of the overview window is provided in SI Fig. S16

A deeper investigation of the local mixing times is possible with the probability density distribution of the local mixing times (Figs. 6 and 10). Those indicate the number of pixels that require a certain amount of time to achieve homogeneity. Additionally, different percentiles are provided in the excel-file, allowing the comparison across replicates.

If smaller maxima or tailing are observed, it indicates that there are areas in the bioreactor which are less thoroughly mixed. When zones with very weak mutual interaction occur, it can be observed that the maxima are far apart. Compared to the non-aerated experiment, the aerated experiment exhibits reduced tailing with a distribution centered around the mean (Fig. 10), whereas the non-aerated case displays a positive (right-sided) skewness (Fig. 6). Furthermore, the aerated case exhibits a third local maximum in the tailing edge of the distribution, suggesting the formation of distinct zones. The representation of the local mixing times in the two-dimensional color-coded chart in combination with the probability density distribution helps to locate zones with poor mixing and draw conclusions about their size and the fluid exchange between the zones.

**Fig. 11:**
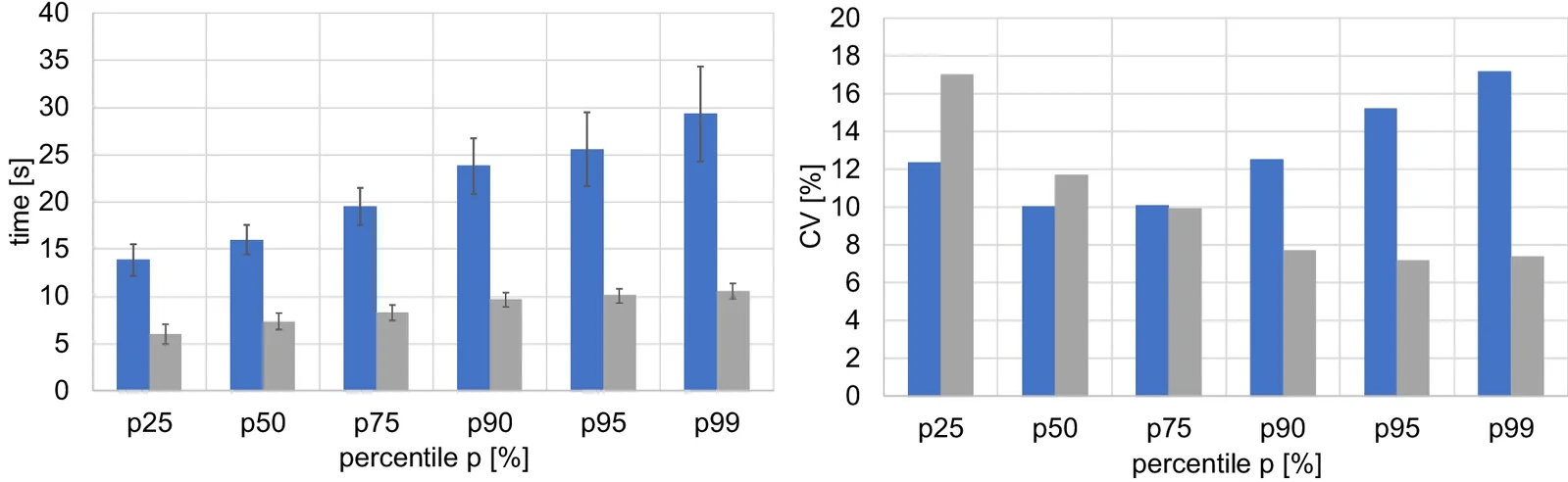
Comparison of mixing times (left) and coefficients of variation (CVs) (right) as a function of the percentiles for the aerated case with 0,55 sLpm (gray) and non-aerated case (blue) with 50 rpm calculated from five replicates

To further quantify the mixing-time field, the percentiles *p*_25_, *p*_50_, *p*_75_ and *p*_95_ were compared for 5 replicates for the aerated experiments and 10 replicates for the non-aerated experiments (Fig. 11). As expected, the mixing time increased with the percentile rank *q*, as a higher threshold requires a larger proportion of pixels to reach the mixed state. The CVs showed values varying from 10.0% (*p*_25_) to 17.2% (*p*_99_) for the non-aerated experiments. In the aerated experiments, the CV values varied from 17.0% (*p*_25_) to 7.2% (*p*_95_). This shows that the datasets have an acceptable, moderate spread. As the local mixing times are calculated pixel-wise, those results show that the data are noise-sensitive, and it is recommended to use a sufficient number of replicates to validate the local mixing results. Furthermore, the flow field may also exhibit heterogeneities.

### Round robin experiment

A round robin test with an SOP is currently being carried out to validate and optimize the automated method for the determination of global and local mixing times (according to the Material and methods section). It is important to emphasize that the results presented herein are preliminary in nature. Although the current data set provides strong trends and validates the core functionality of our method, a definitive assessment requires further extensive testing. Seven different laboratories belonging to members of DECHEMA Working Group “Single-Use Technologies for Bio-Based Applications” are involved in conducting the tests. A double-walled glass bioreactor was chosen for the development of the SOP, as this eliminates some of the additional problems that can be expected with single-use bioreactors (e.g., opaque heating/cooling jackets, less transparent plastic). To ensure direct comparability of the tests carried out in different laboratories, the bioreactor and its components (see Mixing-time experiment section) were exchanged between the laboratories. To configure the experimental settings, different parameters were tested: the injection volume and concentration of the acid added, the aeration and the illumination. The lighting in the selected laboratory is one parameter which could be determined to have an influence on the analysis with the tool. In some laboratories, the direct sunlight was perturbating the evaluation as shadows and reflections occurred. For those laboratories, the usage of a dark tent is recommended. In other laboratories, the position of the bioreactor was not influenced by direct sunlight, and the experiments could be performed without any changes. In particular, the alignment of the bioreactor within the camera frame and a fixed camera position relative to the bioreactor enabled reproducible analysis without distortion. The 800-mm distance between the lens and the shaft allowed for excellent utilization of the entire image, thereby making full use of the camera’s resolution.

**Fig. 12:**
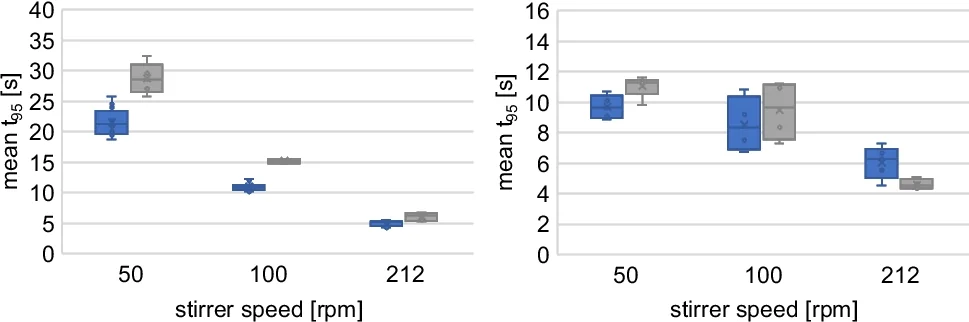
Preliminary results of the round-robin experiment. Comparison of the mean *t*_95_ values for different stirring speeds without aeration (left) and with 0.55 sLpm aeration (right), containing data of 2 out of 7 laboratories, laboratory 1 in blue, and laboratory 2 in gray

The parameters that should be compared (aeration rates and stirring speed) were selected and minor changes regarding the reproducibility were fixed. The injection location was fixed to port N8 of the bioreactor (Fig. 1), the illumination through LED backlight as well as the position of all bioreactor internals and the relative position of bioreactor to camera position were fixed in the SOP. The preliminary results of two laboratories show that the mixing times for the different settings are comparable between the laboratories (Fig. 12). Additionally, it can be concluded that the SOP can produce reliable results. The CV was between 9 and 20%, which corresponds to the values that could be reached by Kraume and Zehner (2001).

A current limitation of the present study is that the inter-operator reproducibility in evaluation has not yet been fully quantified. Further testing regarding the robustness of the evaluation tool is currently carried out. To evaluate the inter-operator reproducibility and ensure the objectivity of the tool, a cross-validation is planned. By analyzing the same benchmark datasets across different operators, we aim to demonstrate the robustness of the algorithm against subjective user inputs, such as the manual definition of the mask and endpoint selection.

## Conclusion

The proposed video-based analysis method offers several advantages over conventional approaches. The use of the final band criterion ensures a physically meaningful definition of mixing time by explicitly requiring temporal stability. Furthermore, the pixel-wise evaluation enables a detailed characterization of spatial heterogeneity, allowing for the identification of slow-mixing regions and the quantification of mixing quality. The method is non-invasive, computationally efficient, and applicable to a wide range of (optically assessable) mixing systems. The combination of global and local metrics provides a comprehensive framework for assessing both mixing speed and spatial uniformity.

Initial experiments showed that the transparent double wall of the glass bioreactor used did not interfere with the measurement. The backlight illuminated the whole bioreactor compartment and allowed a good representation of the bioreactor with the camera. It could be demonstrated that the developed method enables detailed statements about mixing in bioreactors or blending tanks as desired. Differences between mixing in aerated and non-aerated bioreactors can be reliably detected using the developed Python script. Although air bubbles cause noise in the data, experiments with aerated bioreactors can also be evaluated reproducibly. In line with expectations based on the literature, aeration at low stirring speeds in the tested setup led to faster mixing. Critical areas such as “dead” zones can be identified using the two-dimensional color-coded charts of local mixing times that are generated. The inclusion of an ROI enables the possibility to evaluate individual regions, identify compartmentation, and allow problematic regions to be assessed individually.

The preliminary inter-laboratory comparison confirms the reliability of the SOP and its evaluation procedures. The other participating laboratories are currently proceeding with the experiments. Concurrently, a detailed investigation into the operator-dependent steps of the evaluation (such as mask, ROI and end frame selection) is underway to quantify their exact impact on reproducibility. To improve reproducibility in the experimental procedure by varying operators and to transfer the method to single-use bioreactors, the experimental setup was optimized as described in Barth et al. (2026).

The combined analysis of global and local mixing metrics demonstrates that the mixing process cannot be fully characterized by a single scalar mixing time. While the global mixing curve suggests a rapid convergence to the steady state, the spatially resolved analysis reveals persistent inhomogeneities that remain undetected by averaged quantities. This discrepancy highlights the importance of spatial diagnostics, particularly in applications where uniform product quality is critical. Slow-mixing regions may drastically affect reaction yield, selectivity, or product consistency, even if global metrics indicate satisfactory mixing performance.

Despite its advantages, the method is subject to certain limitations. Variations in illumination, optical distortions, or noisy data may influence the results if not properly controlled. Therefore, the mask must be defined to ensure the entire bioreactor volume remains within the unmasked region (especially at the top and bottom), while the stirrers and surroundings of the bioreactor should be excluded. Future work may focus on extending the approach to multi-channel or color-based analyses, as well as incorporating advanced image processing techniques to improve robustness and accuracy.

Overall, the presented methodology provides a powerful and versatile framework for the quantitative analysis of mixing processes (in visibly accessible tanks), with strong potential for both scientific and industrial applications. In particular, this method offers the benefit of rapidly characterizing single-use bioreactors. These systems typically exhibit greater design variability because they can be easily modified and produced using 3D printing technologies.

## Supplementary information

Below is the link to the electronic supplementary material.

ESM 1 (PDF 5.65 MB)

## Data Availability

The authors declare that the data supporting the findings of this study are available within the paper. Should any raw data files be needed in another format they are available from the corresponding author upon reasonable request.

## Abbreviations

*b*: Blue channel of digital image
CFD: Computational fluid dynamics
CV: Coefficient of variation
Δ*t*: Frame duration
DW: Double-walled
*E*(*t*): Mean absolute endpoint deviation of pixel intensities
*E*_0_: Endpoint deviation of the first frame
*ε*: Tolerance
*f*_unmixed_: Unmixed fraction
*G*: Grayscale value
*g*: Green channel of digital image
*I*(*t*): Spatially averaged intensity at time *t*
*I*(*x,t*): Intensity at position *x* and time *t*
*I*_0_: Initial mean intensity corresponding to 0%
*I*_ref_: Final reference mean intensity corresponding to 100%
LED: Light-emitting diode
LES: Large-eddy simulation
*M*_e_(*t*): Endpoint deviation index
*M*_h_(*t*): Homogeneity index
*M*_CV_(*t*): CV reduction index
*µ*(*t*): Mean pixel intensity within the unmasked area
*n*: Smoothing duration
*N*_p_: Total number of pixels
*N*_f_: Total number of frames
Ω: Unmasked area/evaluated spatial domain
*p*_q_: Percentile-based mixing times
*q*: Percentile rank (e.g., 95%)
*Q*_q_: Q^th^ percentile of the distribution of pixel-wise local mixing times
*r*: Red channel of digital image
RANS: Reynolds-averaged Navier–Stokes
ROI: Region of interest
*σ*(*t*): Spatial standard deviation
SI: Supplementary information
sLpm: Standard liters per minute
rpm: Rounds per minute
SOP: Standard operating procedure
SU: Single-use
S(t): Global normalized mixing index
$\overline{S}\left(t\right)$: Smoothed global normalized mixing index
*t*: Time
*t*_mix_: Time at which the deviation of *θ*(*t*) from the final value falls within a tolerance of *ε*
t_95_: Time at which the deviation of *θ*(*t*) from its final value falls within a tolerance of *ε* = 0.05
*t*_mix_*(x*): Time at which the deviation of *θ*(*x*,*t*) from its final value falls within a tolerance of *ε* (local mixing time)
*θ*(*t*): Spatially averaged dimensionless local concentration
*θ*(*x*,*t*): Dimensionless local concentration
UF/DF: Ultrafiltration/diafiltration
