# Supplementary material for: Enhancing reproducibility in mixing time determination of stirred tank reactors via automated analysis and standardized inter-laboratory trials
Source: Appl Microbiol Biotechnol. 2026 Jun 30;110(1):194. doi: 10.1007/s00253-026-13941-8 (PMC13319245; doi:10.1007/s00253-026-13941-8)
Supplement: Supplementary file 1 — (PDF 5.65 MB) [file 253_2026_13941_MOESM1_ESM.pdf]

Supplementary Information:

1. Camera adjustment

The videos were recorded with a spatial resolution of 1920 x 1080 px^2 and a temporal resolution of 60 Hz. In the setup underneath the dark tent, the exposure time was set to 1/160 s and the aperture was set to F8. The camera settings and ISO sensitivity were adjusted so that none of the color states of the indicator are overexposed or underexposed.

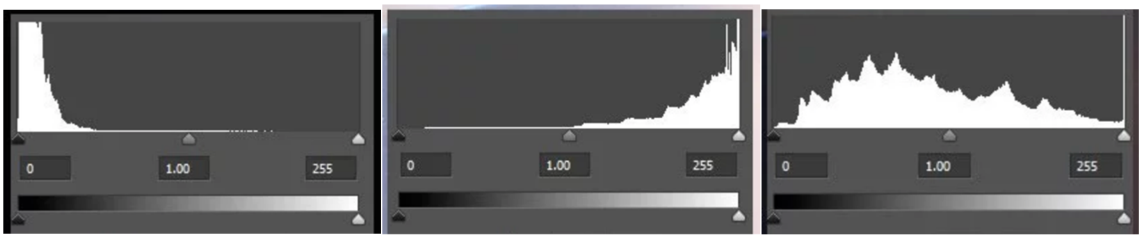

Fig. S1 *Exemplary histograms for the underexposed case, i.e. too much dark areas(left), the overexposed case, i.e. too much light (right) and a balanced histogram (right side)*

2. Screenshots of the mixing time analysis tool

2.1. Setup

In the Mixing Analysis Setup window, the video file can be selected. Different settings can be made. If a batch of videos is processed, an existing mask can be used. Additionally advanced settings are available.

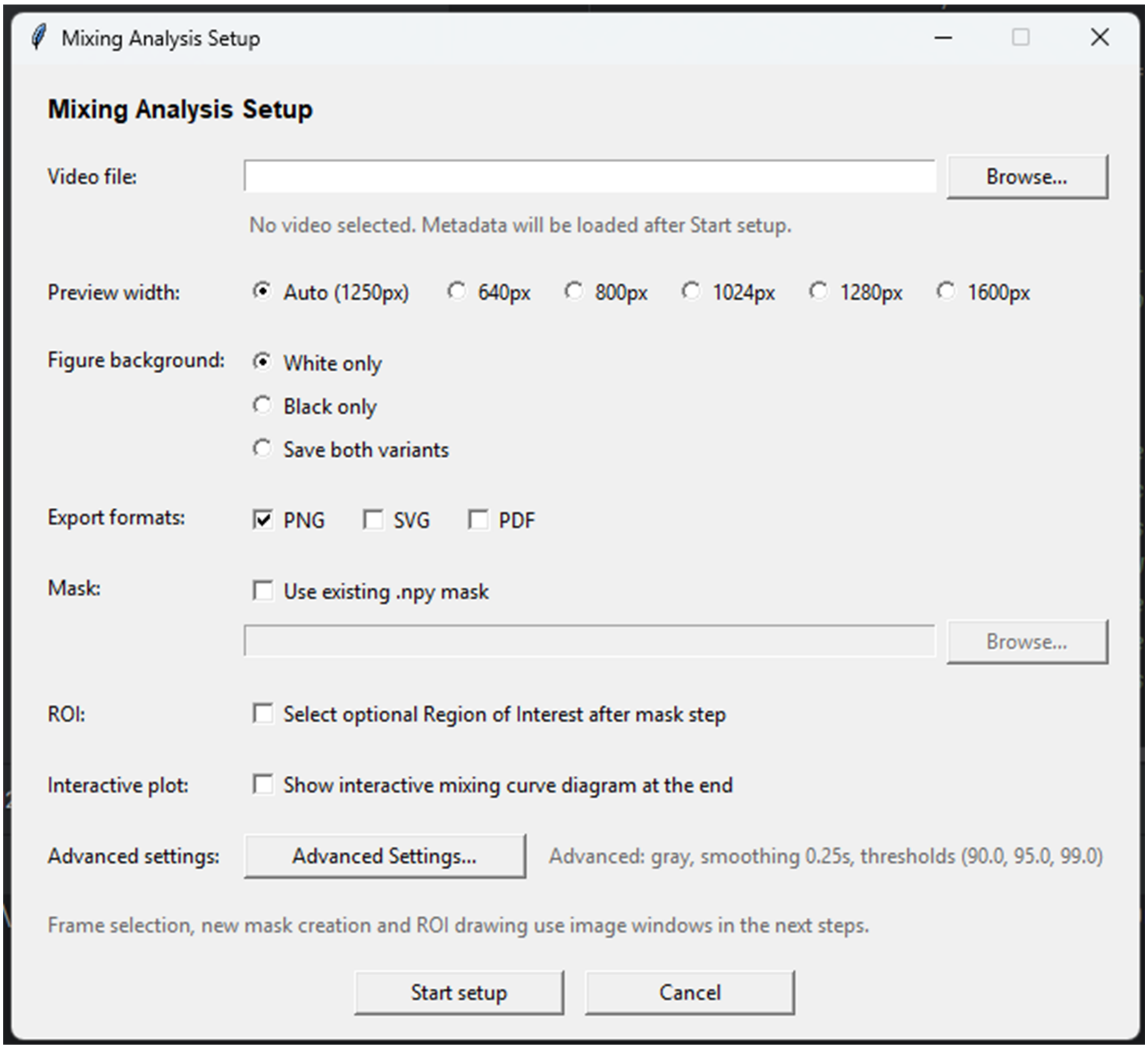

Fig. S2 *Mixing Analysis Setup Window*

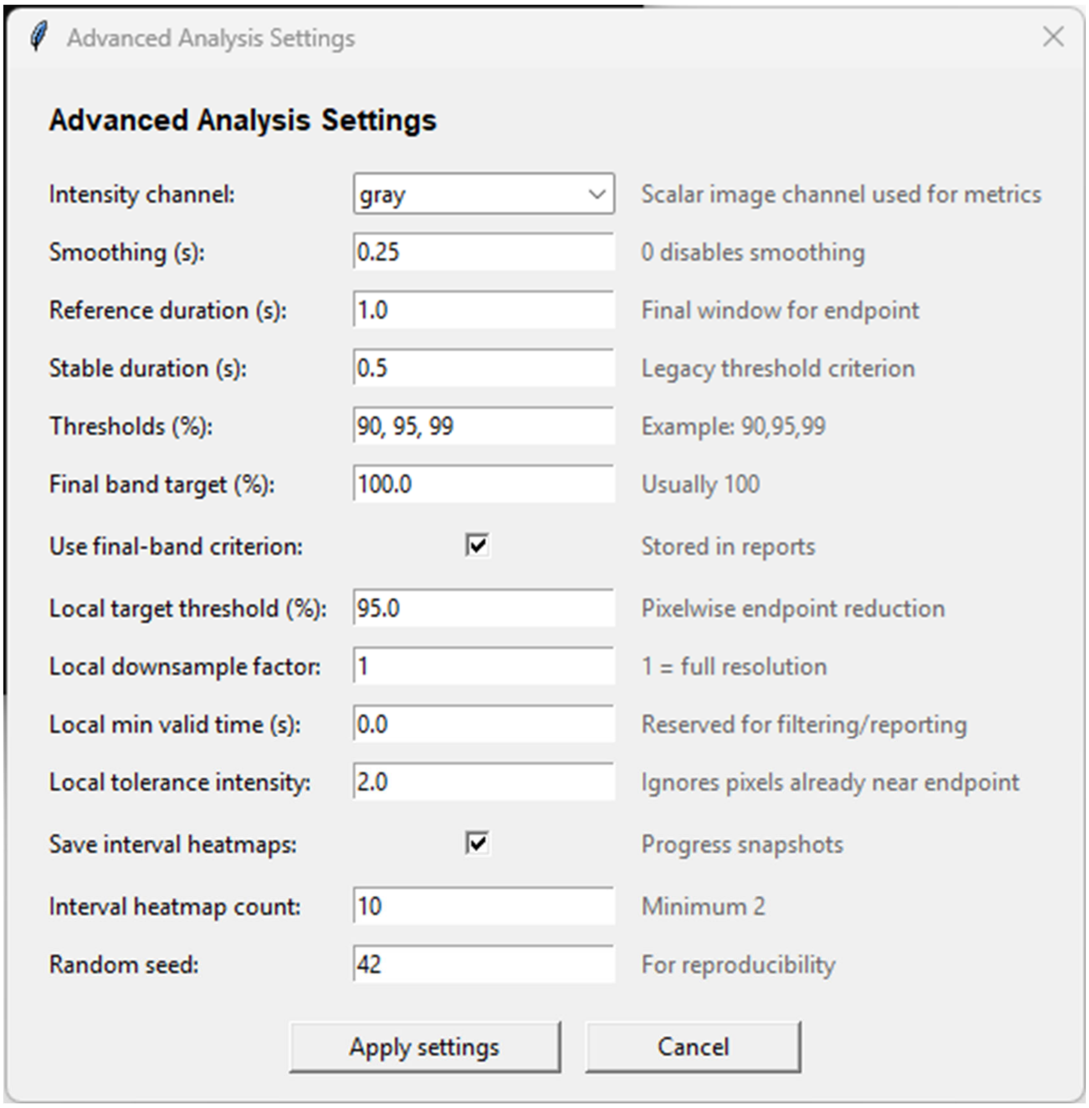

Fig. S3 *Advances settings*

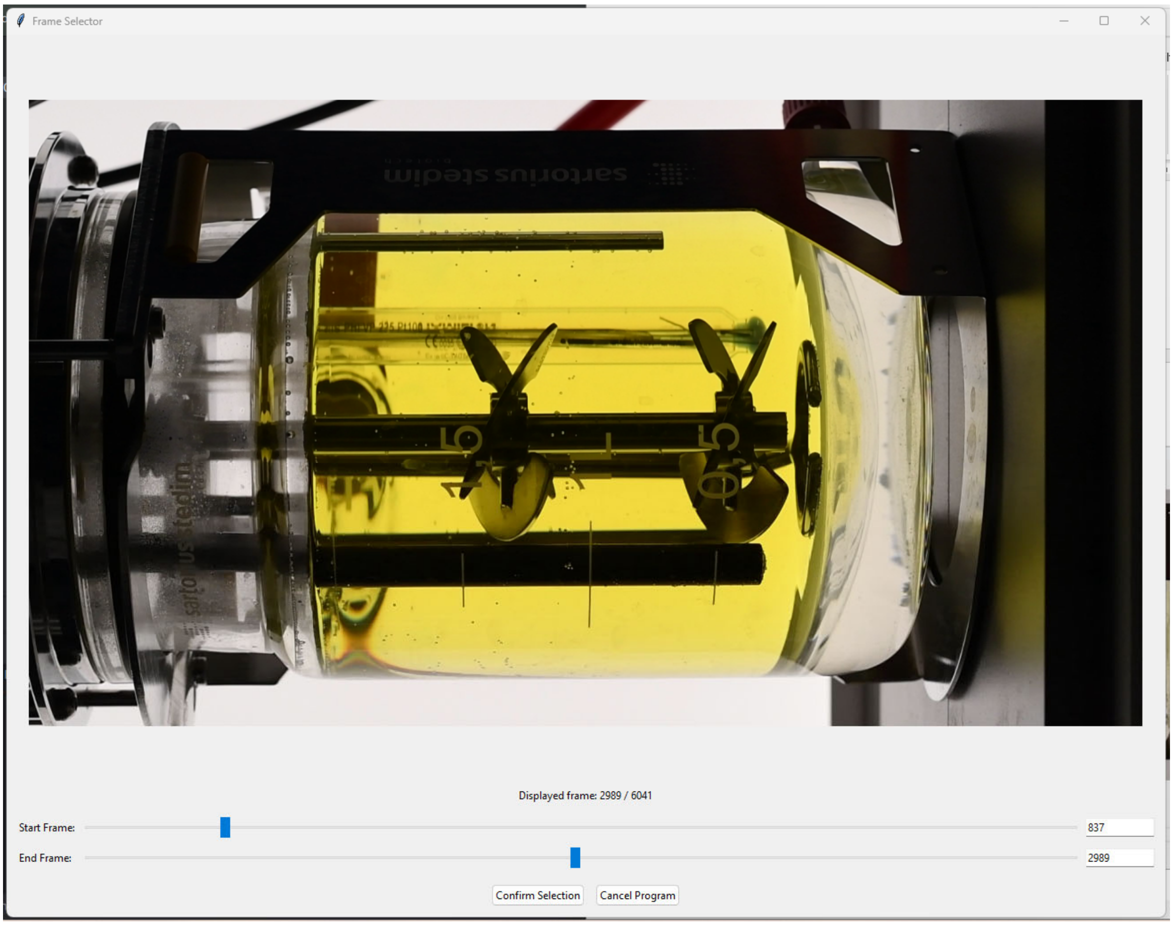

Fig. S4 *Frame selector*

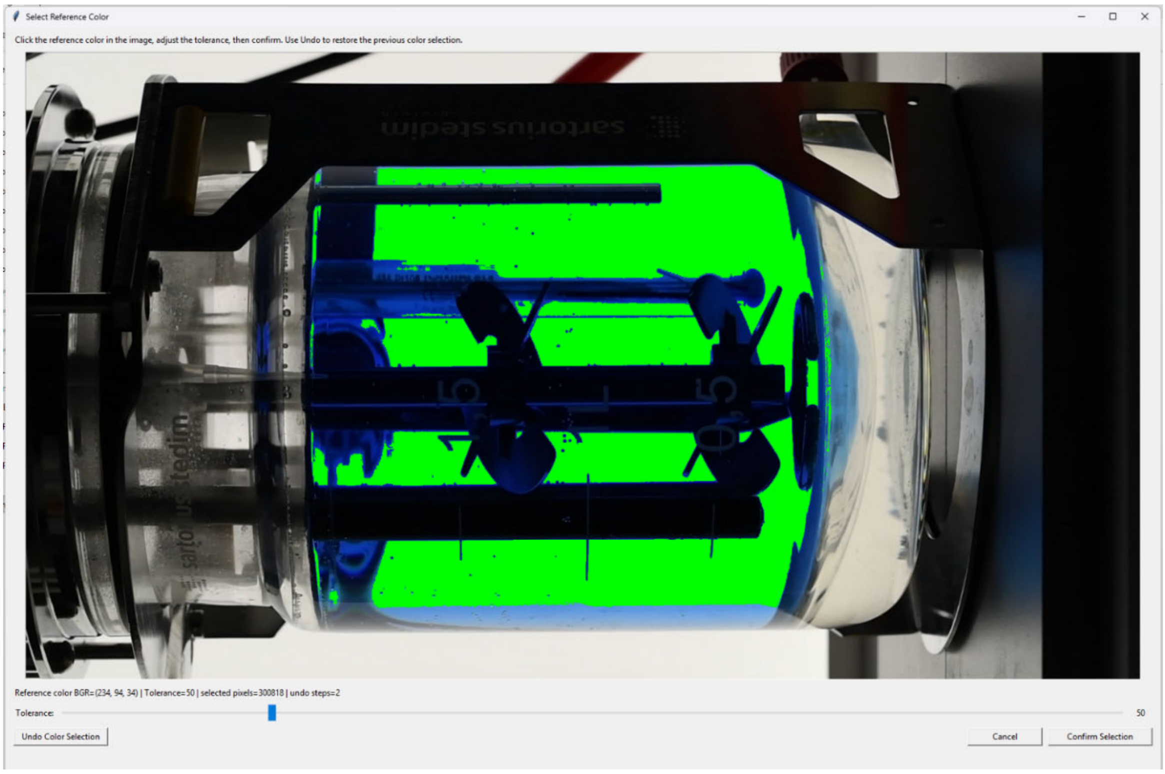

Fig. S5 *Mask selector: first window color reference selector*

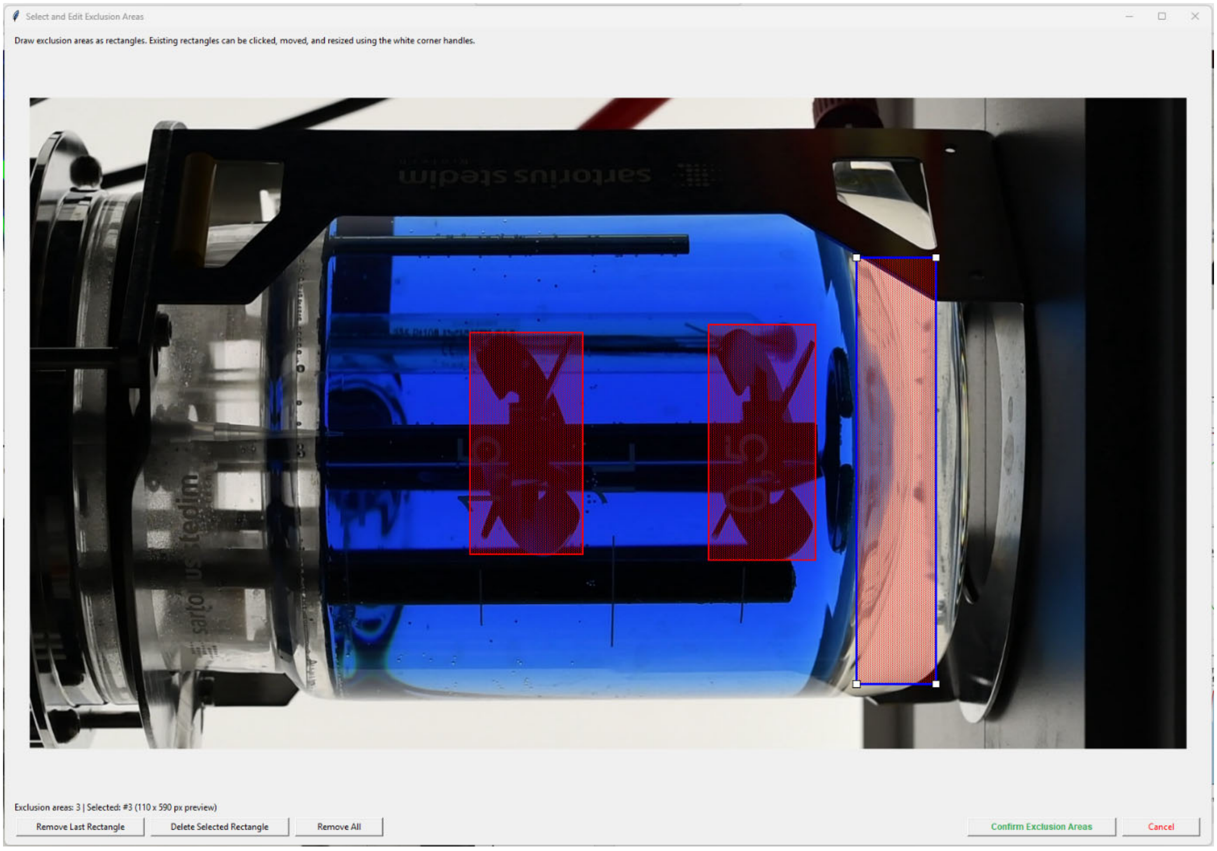

Fig. S6 *Mask selector: second window exclusion area selector*

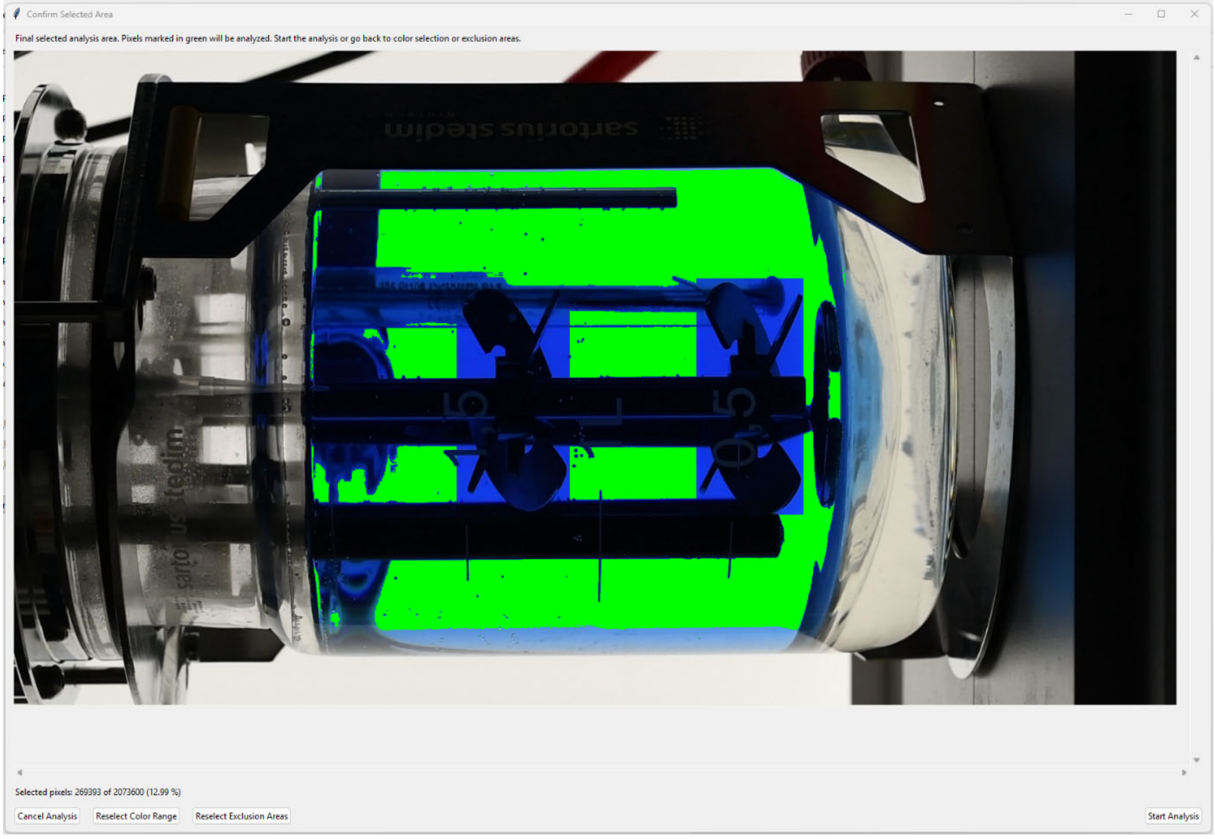

Fig. S7 *Mask selector: third window Confirmation of the masked area*

If the selection of the region of interest (ROI) was selected, in the next step another window is opened, where the ROI can be selected via a blue frame. This step can be confirmed by pressing the enter button.

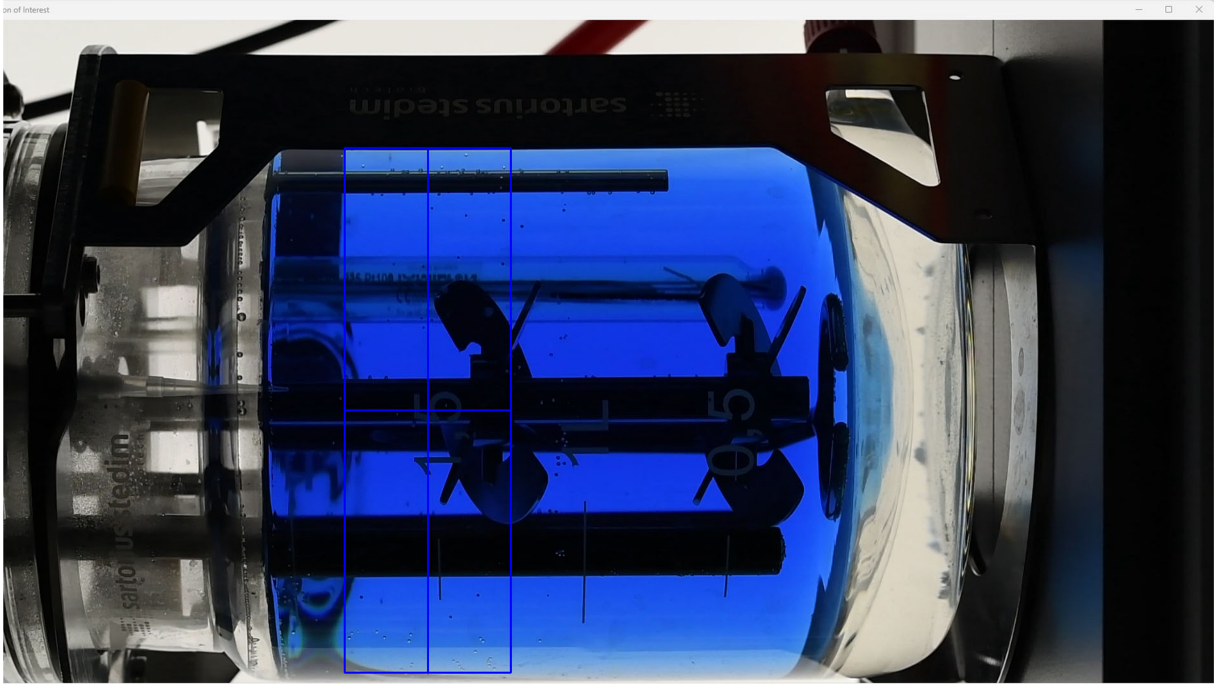

Fig.S8 *Selection of the ROI*

2.2. Analysis

After the mask (and if selected, the ROI) has been selected, the analysis can be started. During the calculations, two progress bars are shown in the command window. Finally the overview window appears to show different graphs.

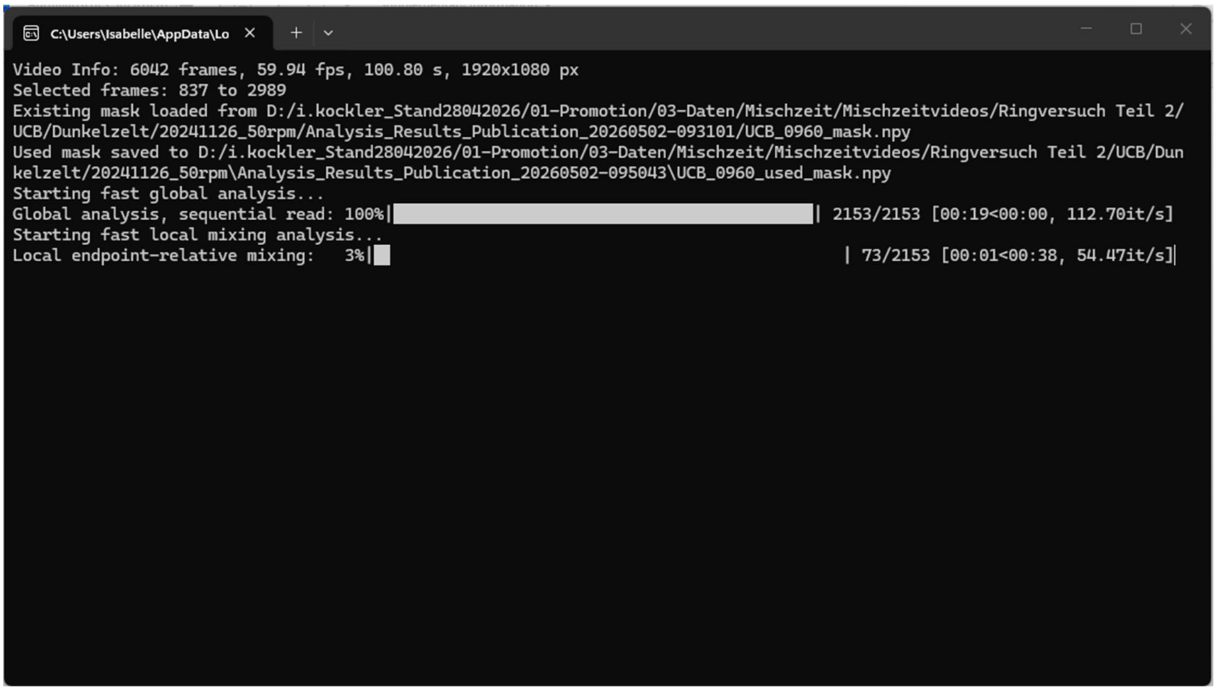

Fig. S9 *Command window, showing the progress*

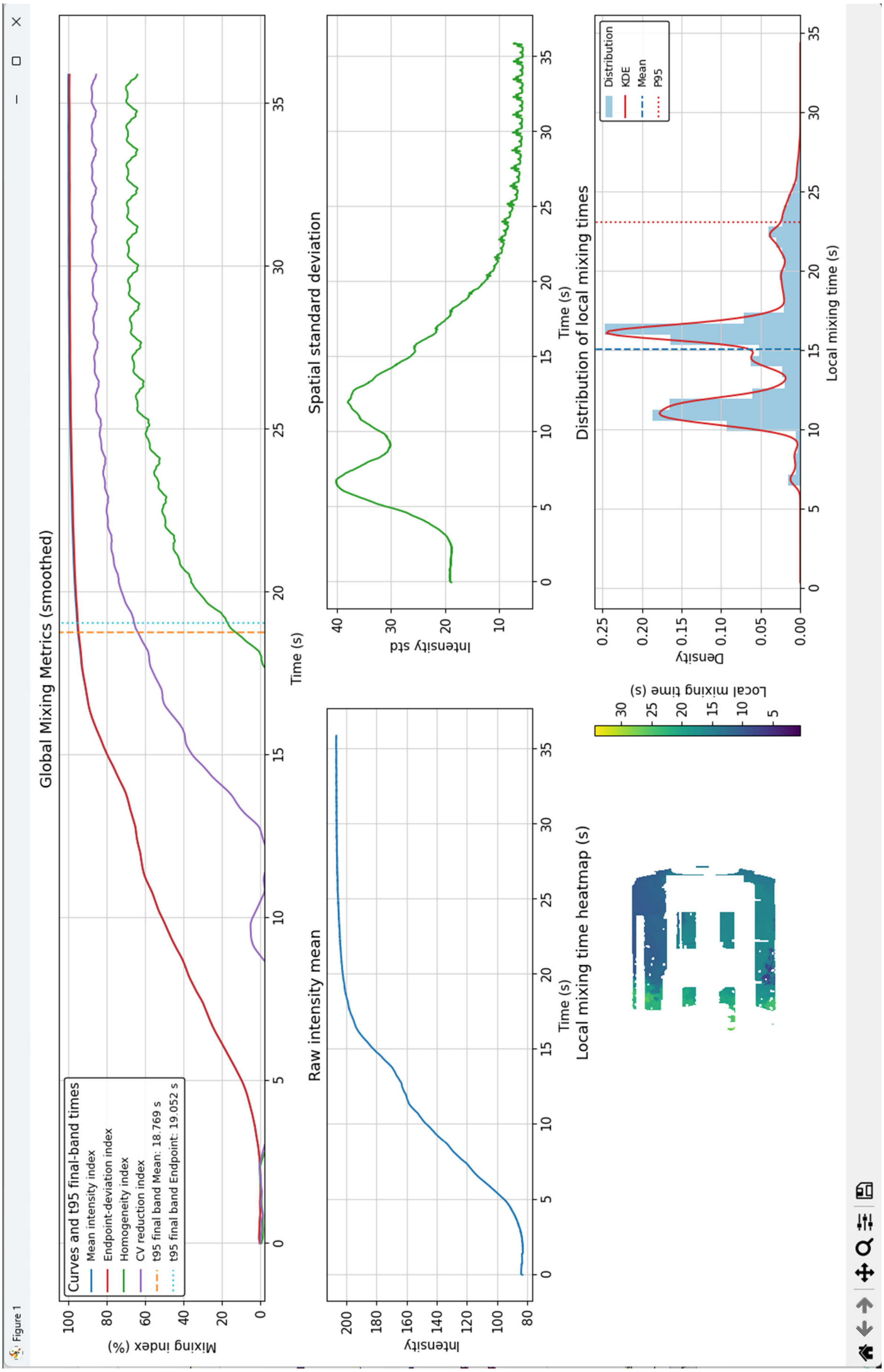

Fig. S10 *Overview of the results*

If the interactive curves were selected in the setup window, another additional window is opened. With the mouse, the cursor can be moved over the x-axis, allowing to see the values of the mixing index to the selected time point.

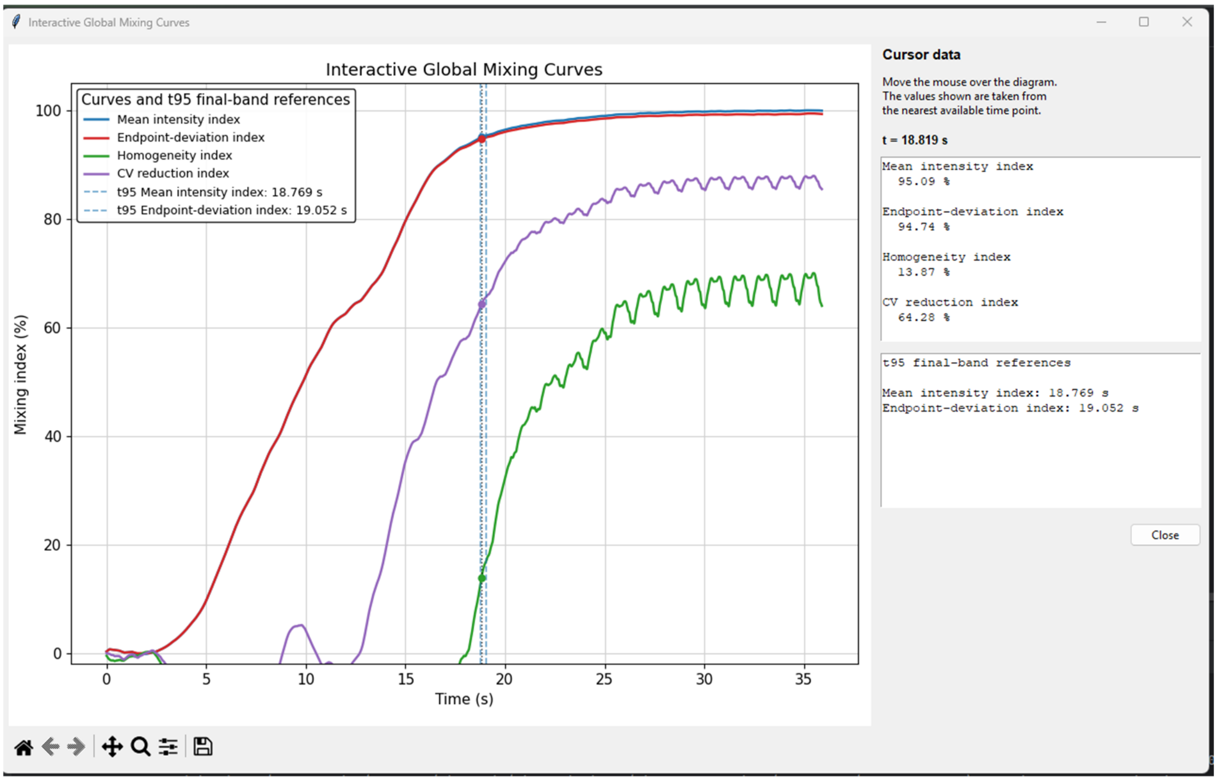

Fig. S11 *Interactive global mixing curves*

3. Results

3.1. Masking without aeration

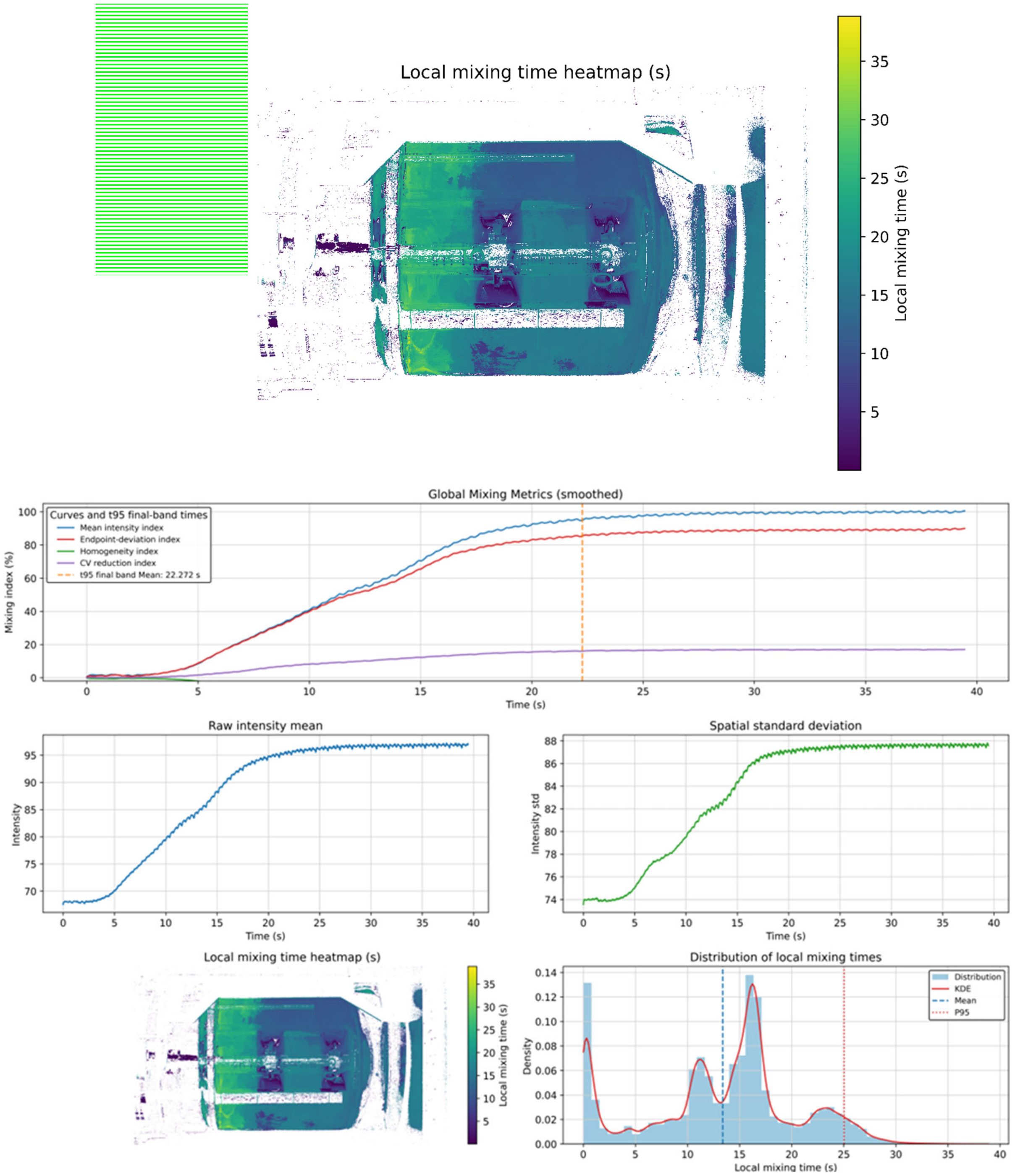

Fig. S12 *Overview window if the mask is deselected and the whole image with surroundings is evaluated.*

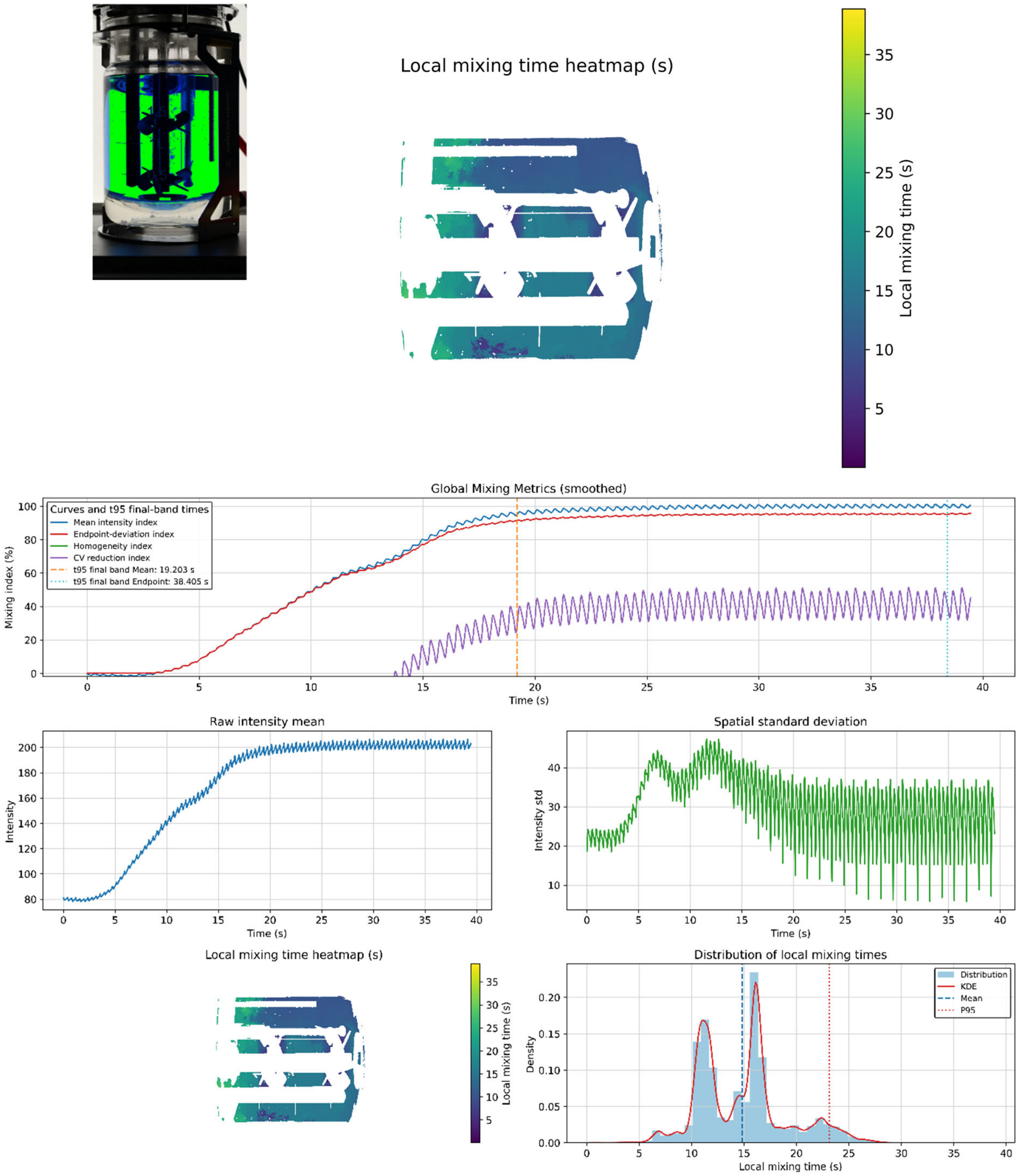

Fig. S13 *Mask selected with the color picker, without any additional changes and no additional exclusions (A), 2D color coded diagram of the local mixing times (B), result of the video analysis (overview window), which shows very sharp fluctuations especially in the diagram showing the standard deviation.*

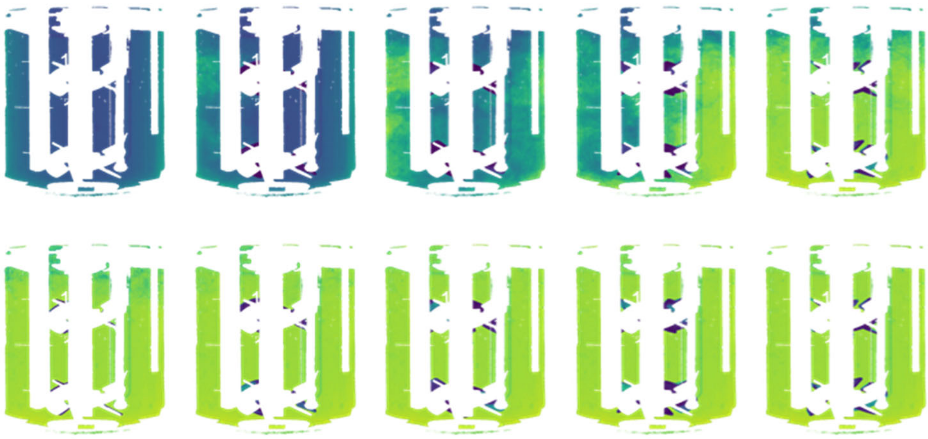

Fig. S14 *Intermediate states of the mixing in the selected area of the bioreactor. The colored part of the image is the investigated area. Due to the rotation of the stirrers, the position changes with the frames. The dark areas around the stirrers show the stirrer position when it differs from the position on the starting frame. The colors vary from violet to blue, explaining the fluctuations in the standard deviation.*

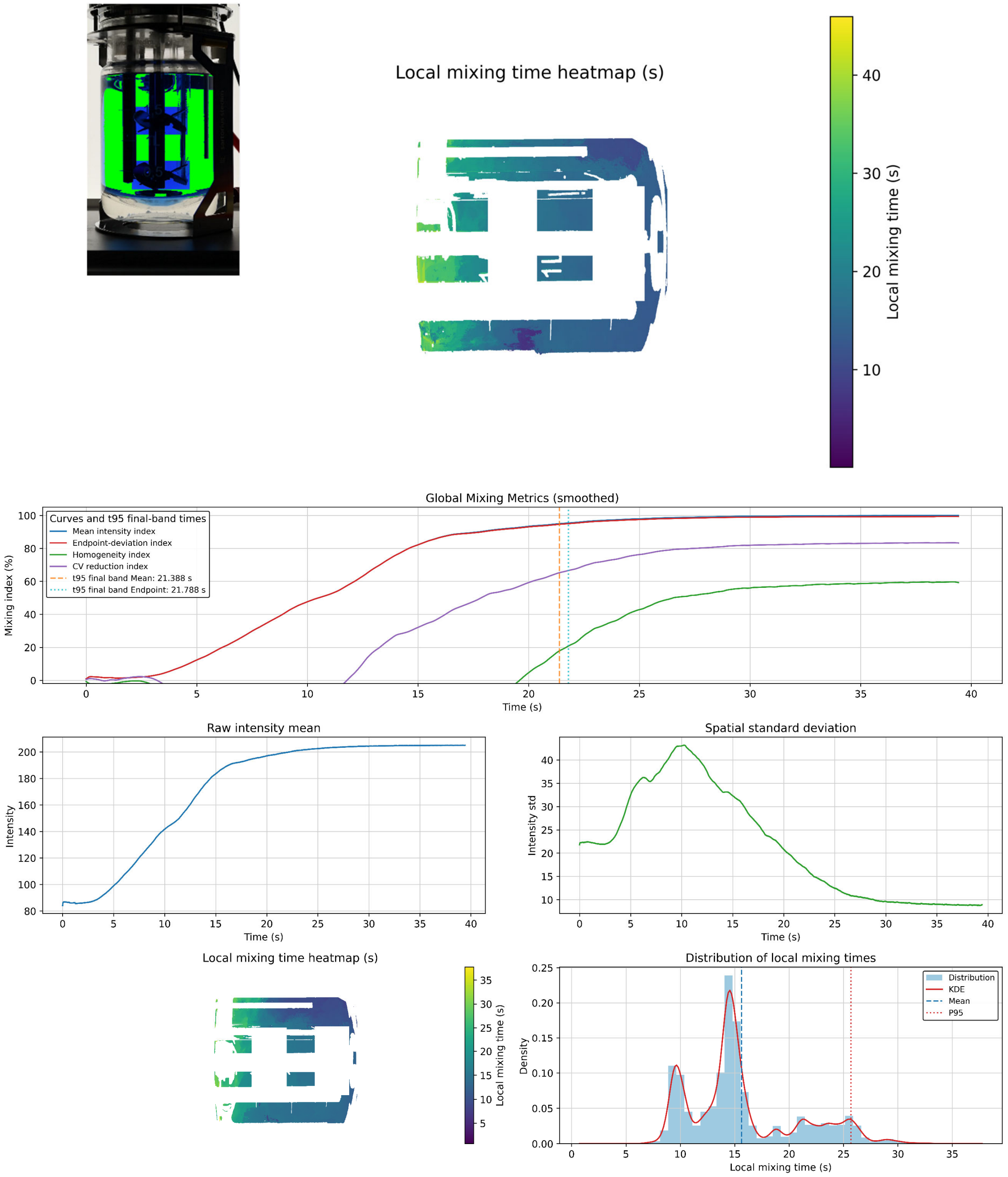

Fig. S15 *Mask selected with the color picker, selection of the full bioreactor volume exclusion of the stirrers and surroundings of the bioreactor (A), 2D color coded diagram of the local mixing times (B), result of the video analysis (overview window), which shows no fluctuations.*

3.2. Masking with aeration

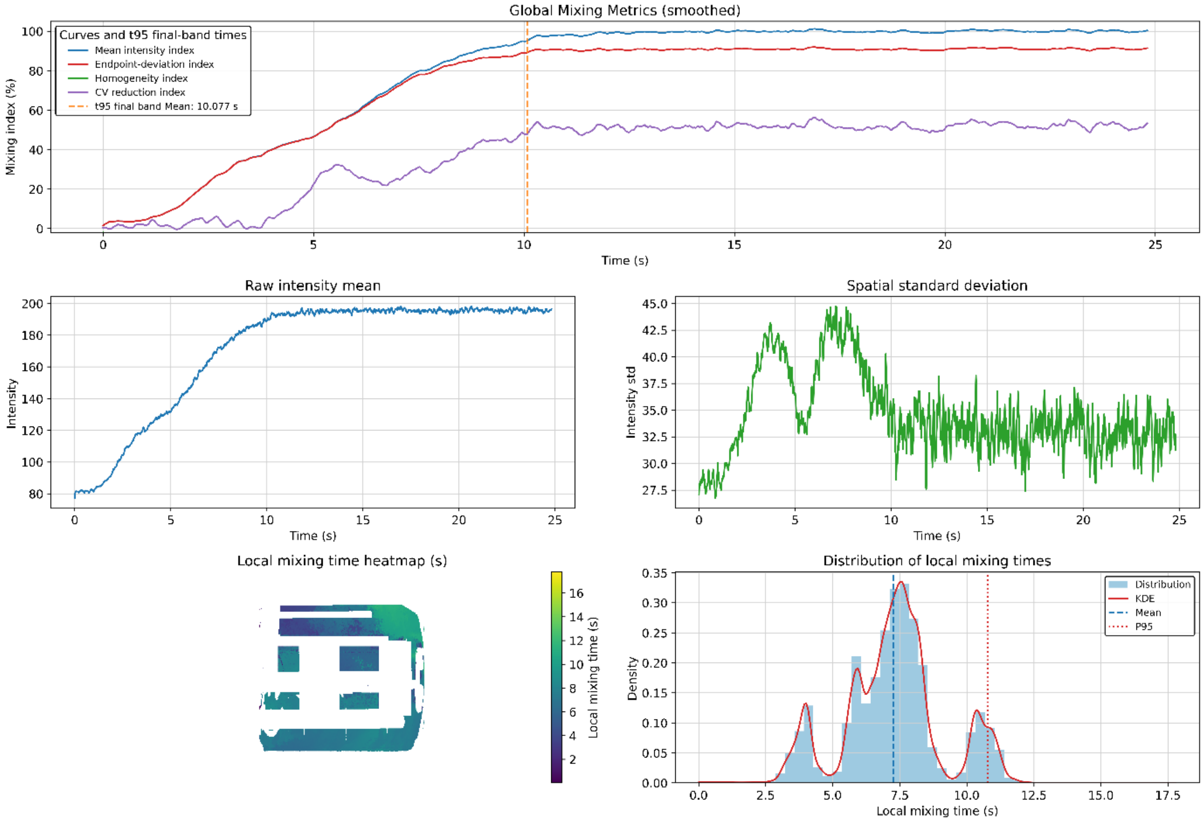

Fig. S16 *Result of the video analysis (overview window) of an aerated video. The mask was selected with the color picker, selection of the full bioreactor volume exclusion of the stirrers and surroundings of the bioreactor in a video without aeration recorded before the start of the aerated experiment.*
